# Intimate partner violence against women living with and without HIV, and the associated factors in Wolaita Zone, Southern Ethiopia: A comparative cross-sectional study

**DOI:** 10.1371/journal.pone.0220919

**Published:** 2019-08-23

**Authors:** Mengistu Meskele, Nelisiwe Khuzwayo, Myra Taylor

**Affiliations:** 1 School of Public Health, Wolaita Sodo University, Wolaita Sodo, Ethiopia; 2 School of Nursing and Public Health, Discipline of Rural Health, University of KwaZulu-Natal, Durban, South Africa; 3 School of Nursing and Public Health, Discipline of Public Health, KwaZulu-Natal, Durban, South Africa; Anglia Ruskin University, UNITED KINGDOM

## Abstract

**Objectives:**

This study aimed to measure the prevalence and associated factors of Intimate Partner Violence (IPV) among women living with and without HIV in Wolaita Zone, Southern Ethiopia.

**Methods:**

A comparative cross-sectional study design was used to interview the 816 women between 18–49 years of age (408 = HIV positive, 408 = HIV negative). Using a multistage sampling technique, participants were recruited from nine health facilities based on probability proportional to the number of clients. After data entry (EpiData version 4.4.2.0) the data were exported to STATA/SE 15 software. Binary and multivariable logistic regression analysis were undertaken and the odds ratio (OR) and 95% confidence interval (CI) are presented.

**Results:**

The lifetime prevalence of IPV among all women was 59.7%, [95% CI: 56.31%-63.05%]. IPV was slightly higher among women living with HIV, 250(61.3%), than those who were HIV negative, 238(58.1%). Lifetime prevalence of emotional violence 413(50.6%), physical violence 349(42.8%), sexual violence 219(26.8%), and controlling behaviours by husbands/partners 489(59.9%) were reported. Associations were found between IPV and controlling behaviour of husband/partner [AOR = 8.13; 95% CI: 4.93–13.42],income [AOR = 3.97; 95% CI:1.81–8.72], bride price payment [AOR = 3.46; 95% CI:1.74–6.87], women’s decision to refuse sex [AOR = 2.99; 95% CI: 1.39–6.41],age group of women [AOR = 2.86; 95% CI:1.67–4.90], partner’s family choosing wife [AOR = 2.83; 95% CI:1.70–4.69], alcohol consumption by partner [AOR = 2.36;95% CI:1.36–4.10], number of sexual partners [AOR = 2.35; 95% CI:1.36–4.09], and if partner ever physically fought with another man [AOR = 1.83; 95% CI:1.05–3.19].

**Conclusions:**

There is a high prevalence of IPV against women both living with and without HIV. Policy priorities should therefore involve males in programs of gender-based violence prevention in order to change their violent behaviour, and interventions are required to improve the economic status of women. Both sexes should be advised to have a single partner and marriage arrangements should be by mutual consent rather than being made by parents.

## Introduction

Intimate Partner Violence (IPV) is increasingly recognized as a serious, worldwide public health concern. Violence Against Women (VAW) occurs in almost all countries, among women and girls of all ages, cultures, races, religions, educational levels and sexual orientation [1]. As a result, in the ever-partnered women, physical or sexual violence or both, by an intimate partner ranged from 15–71% [2]. The global prevalence of physical and or sexual abuse was 30%,[3].The prevalence was highest in the World Health Organization (WHO) African, Eastern Mediterranean and South East Asian regions, where 37% of women reported sexual and or physical violence. The magnitude decreased in high-income countries to 23.2% [3]. The elimination of such violence against women is emphasized in the Sustainable Development Goals (SDG) [4].

IPV is defined as “Any act or omission by a current or former intimate partner which negatively affects the well-being, physical or psychological integrity, freedom, or right to full development of a woman”[5]. IPV is a major public health problem strongly associated with HIV infection among women in Africa and includes different types of violence such as emotional and physical violence and the controlling behaviour by males of their partners.[6].

Recent developments concerning IPV have heightened the need to emphasize the elimination of all forms of violence against women and girls in public and private institutions [4]. Moreover, the issue of IPV against women who are living with HIV is of concern and has received considerable critical attention [7].

Ethiopia is one of the countries with a large burden of Gender-Based Violence (GBV). In the international survey, the lifetime prevalence of sexual violence ranged from 6% (in Japan, Serbia and Montenegro), to 59% in one of the Ethiopian provinces; while physical or sexual violence, or both in Ethiopia has been reported at 54% among women [1]. In addition, a study conducted in Ethiopia by Semahegn, et al.,(2015) showed that the lifetime prevalence of IPV ranged from 20 to 78% [8]. The 2016 Ethiopian Demographic and Health Survey (EDHS) also reported that more than one-third (35%) of ever-married women have experienced physical, emotional, or sexual violence by their partner, and this was supported by a study where 30.4% of ever married women in the southern region of Ethiopia had experienced physical, sexual or emotional violence by their partner [9].

Some studies have identified particular factors associated with IPV, but in Ethiopia IPV has not been well documented amongst women living with and without HIV. Therefore, this study aimed to assess the prevalence and factors associated with IPV among women living with and without HIV, in Southern Ethiopia.

## Materials and methods

### Study area and setting

This study was conducted in Wolaita Zone, one of the 14 Zones in the Southern Nations, Nationalities and People’s Regional State (SNNPR) of Ethiopia. Wolaita is one of the Omotic languages spoken in Southern Ethiopia and is the native language of the Zone. The Amharic language is the official language of the region and the Zone. The capital city of the Zone, Wolaita Sodo, is 330 km south west of Addis Ababa, the capital city of Ethiopia. Currently, Wolaita Zone has a total of 19 health facilities which are providing ART services. The overall HIV prevalence in Ethiopia among men and women aged 15–49 was 0.9%, (CI: 0.7%-1.1%]). It is higher among women than men (1.2% versus 0.6%). However, the prevalence of HIV was lower in Southern Ethiopia, 0.4% [10]. Regarding poverty, Ethiopia is one of the low- income countries and Wolaita Zone is categorized as such. The total population of the Zone was 2,492,887 as estimated on February 8, 2019 [11].

### Design and population

A comparative cross-sectional study design was used to conduct the study among the 19 selected health facilities in Wolaita Zone, from November 2018-December 2018. The target population was all adult women (aged 18–49 years) who were known to be living with HIV, since they had already tested for HIV and were taking Anti-Retroviral Therapy (ART). The comparison group was women without HIV. These were women who were attending health facilities for PMTCT, antenatal care, postnatal care, family planning, and other health services and their HIV sero-status was already documented and it was known that they were HIV negative. No new HIV testing was conducted for this study, since there was available HIV testing information. Women were approached to give their permission to participate.

### Inclusion criteria

There were two groups comprising a) Adult women, aged 18–49 years, who were living with HIV, and using Anti-Retroviral Therapy (ART), b) HIV negative women who were using the different health services mentioned above. The study enrolled women who currently had male partners, whether married or not.

### Exclusion criteria

Women, below 18 and above 49 years, mentally ill or having any other severe illness which hindered the client from being interviewed were excluded. Women excluded from the study could however, also potentially benefit from the research findings.

#### Measurement and operational definition

**Intimate Partner Violence (IPV)**: Is an outcome variable and was measured when the women reported one or more acts of physical, sexual, and/or emotional violence by a current or former male partner, whether cohabiting or not, since the age of 15 years’ [1, 3, 8, 12, 13].

**Physical Violence**: This was measured when women reported at least one experience of a current or former partner who had “ever slapped her, or thrown something at her that could hurt her, pushed or shoved her, hit her with a fist or something else that could hurt, kicked, dragged or beaten her up, choked or burnt her on purpose; threatened her with, or actually used a gun, knife or other weapon against her”[1].

**Sexual Violence**: This was measured when women reported at least one experience of the three sexual violence questions. “Being physically forced to have sexual intercourse against her will, having sexual intercourse because she was afraid of what her partner might do, and being forced to do something sexual she found degrading or humiliating”[1]

**Psychological Violence/emotional violence**: This was measured when the women reported at least one emotional violence item from the four questions listed in the WHO multi-country women and violence study questionnaire. Emotional violence occurs when someone says or does something to make a person feel stupid or worthless [1].

**Controlled behaviour:** This was measured when the women affirmed at least one item of the seven controlled behaviour questions set in the WHO multi-country women and violence study [1].

### Sample size calculation

The sample size was calculated by using the sample size calculator for designing clinical research (http://www.sample-size.net/means-sample-sizeclustered/). Hence, the number of health facilities which are providing ART (clusters = 19), rejecting the null hypothesis (margin of error = 0.05), power = 80%, proportion of women exposed to IPV in South West Ethiopia = 0.415 [14], proportion of women unexposed to IPV = 0.585 (14), within-cluster correlation coefficient = 0.29, confidence interval = 95%. The sample size calculated by using the above formula was adjusted for clustering, and the fixed cluster was 817. The non-response rate and design effect were included in the 408 HIV negative women and the 408 HIV positive women who are using ART.

### Sampling strategy

A multistage sampling technique was used for this study. In Wolaita Zone there are 12 woredas (districts) and three town administrative systems in which 68 health centers and 7 hospitals provide health care services. Though, Wolaita Zone has 68 health centers and seven hospitals, only 19 health facilities (12 health centers and 7 hospitals) which were providing ART in the Zone were included. The reason for this was this study focused on women who were living with HIV and using ART services. In order to compare similar populations, the comparison group, women who were not HIV sero positive were also selected from these 19 health facilities. We randomly selected half of these, namely, nine health facilities from the 19. The health facilities were stratified by the health centers and hospitals. Hospitals were further stratified into governmental and non-governmental. In total 9 health facilities (6 health centers plus 3 hospitals) were randomly selected and included in the study. The sample size for each health facility was allocated based on the probability proportional to the size of their previous six months’ client flow. In order to determine this number, the previous 6 months’ report of the Zonal Health Department was used. Finally, the K^th^ interval (k = N/n), where N = total number of women who were receiving health services in the nine health facilities (N = 5301), was divided by the calculated sample size (n = 817). Every 6^th^ client was interviewed until the desired sample size was reached at each facility.

### Data collection management and storage

The structured questionnaire was adapted from the WHO’s multi-country study on women’s health and domestic violence [5]. The structured questionnaires were prepared first in English and then translated to the local language (Wolaita Donna and Amharic) (S1, S2 and S3 Files). The wording of the questionnaires was adapted to be clear and unambiguous. In order to maintain consistency in the translation with the English version, the questionnaire was back-translated into English by another language expert. The contents of the questionnaire included socio-demographic variables, wealth index, controlling behaviour of partner, emotional, physical, and sexual violence, past violence experienced by husband/partner, and attitudes towards beating one’s partner.

Two days’ training was given to supervisors and data collectors in order to make them familiar with the objectives, the techniques, and the methodology of the research. The training aimed to standardize their interviewing skills and ensure interviews were done in a consistent manner.

Data were collected through interviewer-administered questionnaires in either the Wolaita Dona or Amharic language. Data collection was conducted by eight female health workers. Moreover, four supervisors (BSC/ MPH based on experience) supervised the data collectors.

Before the actual data collection, a pre-test was conducted on 5% of questionnaires in a similar setting but outside the intended study area. In order to maintain the internal consistency of the tool and to determine the reliability of the test, Cronbach’s Alpha was applied. If the alpha was high (≥0.80) the item is considered to be reliable and the test is internally consistent. If the items in the test had a low correlation, rejecting the item that is inconsistent with the rest and retaining the item with the highest average inter-correlation was done via item analysis [15]. The supervisors and the principal investigator, in particular, closely supervised the performance of the data collectors. The completed questionnaires were checked by the supervisors on a daily basis. The raw data has been stored securely and confidentially, and will be kept for five years before being finally discarded appropriately.

### Data analysis

Double data entry was done in EpiData software version 4.4.2.0. The data were then exported to STATA/SE 15, Texas 77845 USA software for cleaning and analyses. The errors identified were corrected after checking the questionnaires. Summary measures such as frequencies, and the regression analysis were computed. Binary and multivariable logistic regression analysis which provided odds ratio and 95% confidence interval were used to identify statistical associations. The variables from the binary logistic regression with (P-value <0.25) were entered one at a time in the multivariable logistic regression to control for possible confounders. Finally, those variables with p-<0.05 were considered as statistically significant. The Hosmer-Lemshow goodness of fit test was done as a post-estimation test by using the STATA command ‘estat gof, group (10)’ and it was not statistically significant (Prob > chi2 = 0.8654).The Receiver Operating Characteristics (ROC) graph was done and the result showed discrimination (0.8796), indicating the model’s ability to discriminate between those subjects who experienced IPV and those who did not. Therefore, we concluded that the model fits. Multicollinearity was investigated using regress and VIF commands, and showed no multicollinearity as none of the variables’ VIF was more than or equal to ten and tolerance was less than 0.1.

### Ethical approval and consent to participate

Ethical approval was obtained from the Biomedical Research Ethics Committee (BREC) from the University of KwaZulu-Natal (BREC Ref No: BE387/18) (S4 File). Ethical clearance was also obtained from the Institutional Review Board of Wolaita Sodo University under Ref No: WSU15/04/147 (12 Sep. 2018) (S5 File). Formal permission was obtained from the health departments of Wolaita Zone and the respective health centers and hospitals where the study was conducted. The information regarding informed consent was explained to the participants in their own language. Respect for the person (autonomy) and protection of vulnerable participants were maintained. The purpose of the research, and the expected duration for the participant to complete the interview/questionnaire (25–30 minutes) was communicated. Referral for psychological support was made for some individuals. This study maintained the confidentiality of the participants; their name was not written on any result (anonymity was ensured). The participant had the right to withdraw or to interrupt their participation at any time without penalty or loss of benefits. We obtained the written consent from each of the participants.To maintain complete privacy, only one woman at a time in a private room or place was interviewed, except for children under age two years.

## Results

### Socio demographic characteristics of women living with and without HIV

A total of 816 women (408 HIV positive and 408 HIV negative) participated in this study. Over half of the women living with HIV, 238 (58.3%), were under the age of 29–39 years. Nearly half, 190 (46.57%) of the women without HIV were in the age group 18–28 years. More than three-quarters of women 324 (79.41) living with HIV were living in urban areas, as were 285(69.85) of HIV negative women. The majority of women who were living with HIV193 (47.3%) had primary education (grades 1–8), whereas the majority of women without HIV 154(37.75%) had secondary education (grades 9–12). The majority, 371(90.93%) of HIV negative women were currently married or lived with a man, whereas, among HIV positive women there were 252 (61.76%) with partners. Many of the HIV positive women were widowed 59 (45.38%). Bride price had been paid for 297 (90%) HIV negative women, while among HIV positive women the figure was lower 191(71%). Regarding the wealth index, many of the HIV positive women, 165 (40.44%) were poor and few of them, 97 (23.77%) had a high wealth index, whilst many of HIV negative women had a higher wealth index 156 (38.24%) and relatively lower poverty, 118 (28.92%). Other than ethnicity, HIV status and a trend for differences in religion there were the significant socio-demographic differences between the HIV positive and negative respondents (Table 1).

**Table 1:** Socio demographic characteristics of Women living with and without HIV in Wolaita Zone, Southern Ethiopia, from November-December 2018.

Characteristics (n = 816)	Women HIV Positive (n = 408)		Women HIV Negative(n = 408)		All women (816)	P-value
	Frequency	Percent (%)	Frequency	%	Number (%)	
**Age**						P<0.001
18–28	119	29.20	190	46.57	309(37.9)	
29–39	238	58.30	193	47.3	431(52.8)	
40–49	51	12.50	25	6.13	76(9.3)	
**Residence**						
Urban	324	79.41	285	69.85	609(74.6)	P<0.001
Rural	84	20.59	123	30.15	207(25.4)	
**HIV sero-status (HIV+ vs HIV-)**	250	61.27	237	58.09	816(100)	P = 0.354
Women education						
No Education	118	28.98	65	15.93	183(22.4)	P<0.001
Primary Education (1–8)	193	47.30	139	34.07	333(40.8)	
Secondary (9–12 Grade)	75	18.38	154	37.75	229(28.1)	
Higher (Above 12)	22	5.39	50	12.25	71(8.7)	
**Current marital status**						
Currently married or lived with a man	252	61.76	371	90.93	623(76.4)	P = 0.004
Living with a man or not married or having a regular partner	24	5.88	10	2.45	34(4.2)	
Not currently married or living with a partner	132	32.35	27	6.62	159(19.4)	
**Payment of bride price**						
All paid	191	71	297	90	488(81.5)	
Partially paid or Non paid	78	29	33	10	111(18.5)	P<0.001
**Religion**						
Orthodox	183	44.85	135	33.09	318(40.0)	P = 0.073
Muslim	19	4.66	16	3.92	35(4.3)	
Protestant	178	43.63	207	50.74	385(47.2)	
Catholic	15	3.68	10	2.45	25(3.1)	
Traditional	13	3.19	1	0.25	1(0.1)	
Apostolic	-	-	39	9.56	52(6.3)	
**Ethnicity**						P = 0.593
Wolaita	337	82.6	350	85.78	687(84.2)	
Amhara	27	6.62	16	3.92	43(5.3)	
Gurage	12	2.94	21	5.15	33(4.0)	
Others	32	7.84	21	5.15	53(6.5)	
**Occupation of women**						
House wife	212	51.96	192	47.06	404(49.5)	P<0.001
Trader	67	16.42	66	16.18	133(16.3)	
Student	21	5.15	52	12.32	73(9.0)	
Government or NGO employ	41	10.05	87	21.32	128(15.7)	
Daily labourer	62	15.20	10	2.45	72(8.8)	
Others	5	1.23	1	0.25	6(0.7)	
**Wealth Index**						
Poor	165	40.44	118	28.92	283(34.7)	P<0.001
Middle	146	35.78	134	32.84	280(34.3)	
High	97	23.78	156	38.24	253(31.0)	

### Controlling behaviours by an intimate partner against women in Wolaita Zone

If the women reported at least one of the seven controlling behaviour questions listed in Table 2 below, then the woman was considered as reporting controlling behaviour. The composite prevalence of controlled behaviour among HIV positive women was thus reported by more than half of the women surveyed, 227 (55.64%), 95% CI (50.80%-60.49%), while nearly two-thirds of HIV negative women, 262(64.22%), 95% CI (59.54%-68.89%), experienced controlled behaviour. The overall prevalence of controlled behaviour among both groups was 489(59.93%) (Table 2)

**Table 2:** Controlling behaviours by an intimate partner against women living with and without HIV in Wolaita Zone, Southern Ethiopia, November-December 2018.

Characteristics (n = 816)	Women HIV positive	Women HIV negative	All women	P-value
	Yes, # (%)	Yes, # (%)	Yes, # (%)	p<0.001
Tries to keep you from seeing your friends	127(31.13)	130(31.86)	257 (31.50)	p<0.001
Tries to restrict contact with your family of birth	110(26.96)	110(26.96)	220 (26.96)	p<0.001
Insists on knowing where you are at all times	169(41.42)	188(46.08)	357(43.75)	p<0.001
Ignores you and treats you indifferently	80(19.61)	35(8.58)	115(14.09)	p<0.001
Gets angry if you speak with another man	153(37.50)	185(45.34)	338(41.42)	p<0.001
Is often suspicious that you are unfaithful	99(24.26)	47(11.52)	146(17.89)	p<0.001
Expects you to ask his permission before seeking health care for yourself	135(33.09)	187(45.83)	322(39.46)	p<0.001
Controlled behavior (composite)	227(55.64)	262(64.22)	489(59.93)	p<0.001

### Magnitude of emotional violence in Wolaita Zone, Ethiopia

**Fig 1:**
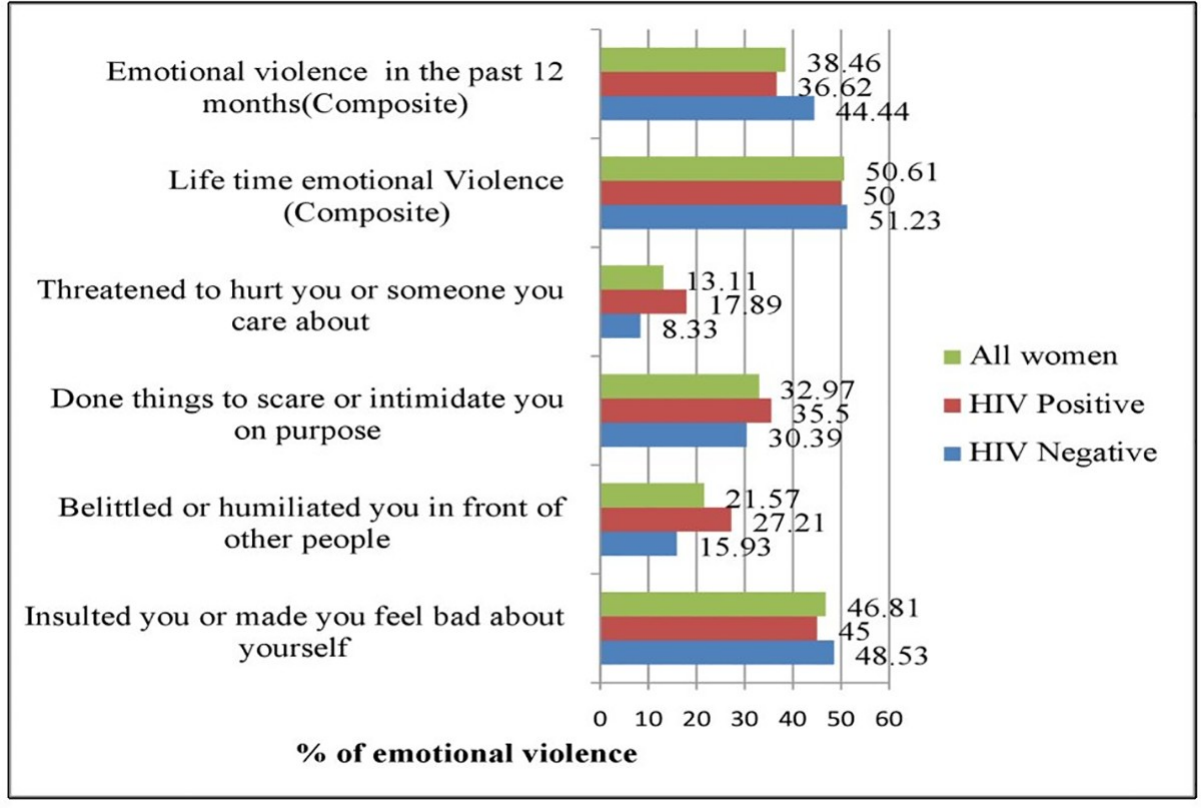
Emotional violence against women in Wolaita Zone, November-December 2018.

If women reported at least one of the four emotional violence questions in Fig 1 below, they were considered as having experienced emotional violence. Accordingly, half of the women living with HIV, 204(50%), [95% CI: 45.13%-54.87%] had experienced lifetime emotional violence, with a similar proportion, 209(51.23%), [95% CI: 46.35%-56.10%] found among HIV negative women. The overall lifetime prevalence of emotional violence among all women (n = 816) was 413(50.61%).

Of the total number of participants who had experienced emotional violence in the past 12 months, (n = 60), 22(36.62%) [95% CI: 24.11–49.22] were among women living with HIV, whereas among HIV negative women (n = 18), there were 8(44.44%), [95% CI: 19.02–69.87]. The overall prevalence of emotional violence in the past 12 months among all 78 women was 30(38.46%) (Fig 1).

### Prevalence of intimate partner violence in Wolaita Zone, Ethiopia

Lifetime physical violence among women living with HIV (n = 408) and without HIV (n = 408) was respectively 185(45.34%) [95% CI: 40.49%-50.19%] and 164(40.20%) [95% CI: 35.42%-44.97%]. Lifetime sexual violence among women living with HIV and without HIV was respectively 114(27.94%) 95% CI: 23.57%-32.31%] and 105(25.74%) [95% CI: 21.48%-29.10%].

The lifetime prevalence of physical and sexual violence among all the women interviewed (n = 816) was respectively 349(42.77%) [95% CI: 39.37%-46.17%] and 219(26.84%) [95% CI: 23.79%-29.88%].

The lifetime prevalence of IPV (women who had reported at least one incident from physical, sexual, and emotional/psychological violence) among all women surveyed (n = 816) were 487(59.68%), [95% CI: 56.31%-63.05%]. Although, the prevalence of IPV was a little higher among women living with HIV, 250(61.27%), [95% CI: 56.53%-66.02%] than women who were HIV negative, 238(58.09%), 95% CI: 53.28%-62.90%], there were no statistically significant differences (P = 0.354)

**Fig 2:**
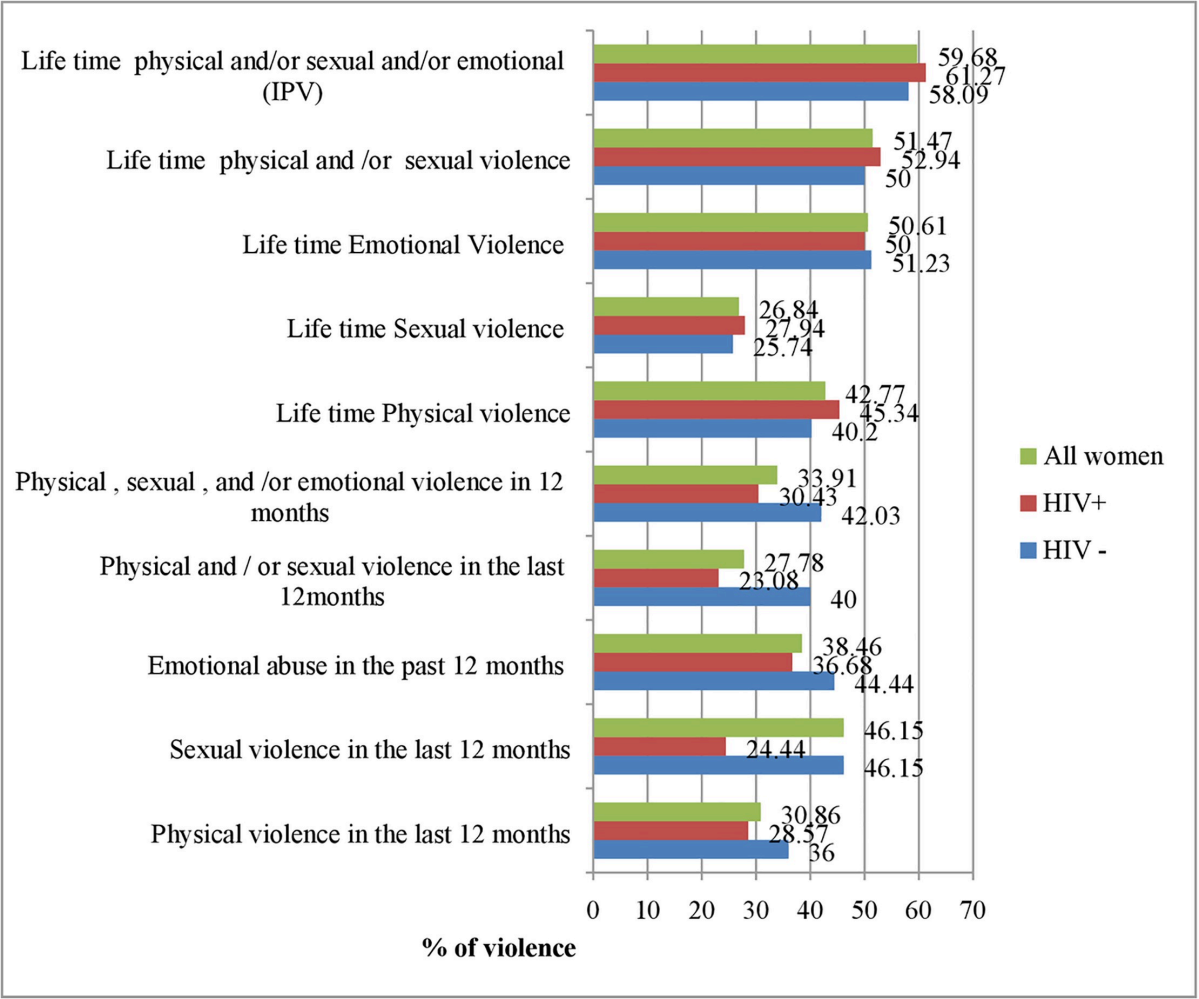
Prevalence of intimate partner violence in Wolaita Zone, Ethiopia, November-December 2018.

In the 12 months preceding the survey, the prevalence of IPV (physical, sexual, emotional /psychological violence) among all women, n = 230, was 78(33.91%). The physical violence in 12 months among all women, n = 81, was 25(30.86%), 95% CI: 20.59%-41.14%] while sexual violence in the same period amongst all the women, n = 71, was 23(32.39%), 95% CI: 21.24.43.55%] **(**Fig 2).

### Prevalence of lifetime physical and sexual violence among women in Wolaita Zone

Different features of physical and sexual violence were identified among women living with and without HIV in Wolaita Zone. Among the features of physical violence, more than one-third of the partner slapped women or thrown something at their partner that could hurt them among women living with HIV, 163(39.95%), while such figure was lower among women living without HIV, 151(37.01%). Moreover, some features of physical violence like male partner choked or burnt their spouse/ partner on purpose was relatively one of the lowest physical violence types among both of the women living with HIV, 9(2.21%), and also the women living without HIV, 4(0.98%), in Wolaita Zone.

Regarding the features of sexual violence, their current husband/partner or any other partner ever physically force women to have sexual intercourse when they did not want to practice was more common among women living with HIV, 86(21.08%), than women who were living without HIV, 66(16.18%). Similarly, women’s male partner or any other partner ever forced them to do something sexual that the women found degrading or humiliating was more common among HIV positive women, 57(13.97%) than their counterpart of women living without HIV, 33(8.09%) (Table 3).

**Table 3:** Prevalence of lifetime physical and sexual violence among women living with and without HIV in Wolaita Zone, Ethiopia, November-December 2018.

Characteristics of physical violence	Women HIV+ (N = 408); Number (%)	Women HIV- (N = 408); Number (%)	All women (N = 816); Number (%)	P-value
Slapped you or thrown something at you that could hurt you	163(39.95)	151(37.01)	314(38.48)	p<0.001
Pushed you or shoved you or pulled your hair	115(28.19)	90(22.06)	205(25.12)	p<0.001
Hit you with his fist or with something else that could hurt you	96(23.53)	62(15.20)	158(19.36)	p<0.001
Kicked you, dragged you or beat you up	95(23.28)	68(16.67)	163(19.98)	p<0.001
Choked or burnt you on purpose	9(2.21)	4(0.98)	13(1.59)	P = 0.003
Threatened to use or actually used a gun, knife or other weapon against you	24(5.88)	11(2.70)	35(4.29)	p<0.001
**Characteristics of Sexual Violence**				
Your current husband/partner or any other partner ever physically force you to have sexual intercourse when you did not want to	86(21.08)	66(16.18)	152(18.63)	p<0.001
Had sexual intercourse which you did not want to because you were afraid of what your partner or any other partner might do	93(22.79)	88(21.5)	181(22.18)	p<0.001
Your partner or any other partner ever forced you to do something sexual that you found degrading or humiliating	57(13.97)	33(8.09)	90(11.03)	p<0.001

### Women’s attitudes towards partner beating

In order to better understand the attitudes of women they were questioned the reasons why a man would / could hit/beat his wife. The prevalence of an overall positive attitude towards wife beating among all women was 393 (48.16%).This attitude towards wife beating was somewhat higher among HIV negative women 211(51.72%) than women living with HIV, 182 (44.61%). However, the women’s positive attitude towards wife-beating regarding a refusal of sexual relations in circumstances such as sickness etc. was higher among HIV positive women, 352(86.27%) than those HIV negative, 306 (75%). The overall prevalence was 658 (80.64%) among both groups of women who reported a positive attitude towards wife-beating as a result of the wife’s refusal to have sexual relations in some circumstances such as sickness etc.,(Table 4)

**Table 4:** Women’s attitudes towards partner beating in Wolaita Zone, Ethiopia, November-December 2018.

Characteristics wife beating		HIV Positive Frequency (%)	HIV Negative Frequency (%)	All women	P-value
When she does not complete her household work to his satisfaction	Yes	111(27.21)	89(21.81)	200(24.51)	p<0.001
	No	297(72.79)	319(78.19)	616(75.49)	
When she disobeys him	Yes	137(33.58)	164(40.20)	301(36.89)	P = 0.001
	No	271(66.42)	244(59.80)	515(63.11)	
When she refuses to have sexual relations with him	Yes	101(24.75)	120(29.41)	221(27.08)	P = 0.008
	No	307(75.25)	288(70.59)	595(72.92)	
When she asks him whether he has other girlfriends	Yes	104(25.49)	88(21.57)	192(23.53)	P = 0.003
	No	304(74.51)	320(78.43)	624(76.47)	
When he suspects that she is unfaithful	Yes	128(31.37)	88(21.57)	216(26.4)	P = 0.006
	No	280(68.63)	320(78.43)	600(73.53)	
When he finds out that she has been unfaithful	Yes	149(36.52)	165(40.44)	314(38.48)	P = 0.024
	No	259(63.48)	243(59.56)	502(61.52)	
Attitude towards wife beating (composite)	No wife beating	226(55.39)	197(48.28)	423(51.84)	
	Beat wife	182(44.61)	211(51.72)	393(48.16)	
Characteristics refusal to have sex					
When she doesn’t want to	Yes	241(59.07)	137(33.58)	378(46.32)	P = 0.004
	No	167(40.93)	271(66.42)	438(53.68)	
When he is drunk	Yes	256(62.75)	157(38.48)	413(50.61)	p<0.001
	No	152(37.25)	251(61.52)	403(49.39)	
When she is sick	Yes	319(78.19)	271(66.42)	590(72.30)	p<0.001
	No	89(21.81)	137(33.58)	226(27.70)	
When he mistreats her	Yes	296(72.55)	204(50)	500(61.27)	p<0.001
	No	112(27.45)	204(50)	316(38.73)	
When she suspects he has extra-marital sexual relations with another woman	Yes	293(71.81)	207(50.74)	500(61.27)	p<0.001
	No	115(28.19)	201(49.26)	316(38.73)	
When she suspects her husband has an STI/HIV	Yes	293(71.81)	235(57.60)	528(64.71)	p<0.001
	No	115(28.19)	173(42.40)	288(35.29)	
Attitude towards refusal of sexual relation(Composite score)	Unable to refuse sex	56(13.73)	102(25)	158(19.36)	p<0.001
	Can refuse sex	352(86.27)	306(75)	658(80.64)	

### Factors associated with intimate partner violence against women living with and without HIV in Wolaita Zone, Ethiopia

To identify factors associated with IPV, the independent variables were analyzed in bivariate and multivariable logistic regression models. A total of 23 variables were included in the multivariable logistic regression model. Women who were in the age group 29–39 years were 2.86 times more likely to experience IPV than those in the age group 18–28 years, [AOR = 2.86; 95% CI: 1.67–4.90]. Women with low-income were nearly four times more likely to have experienced IPV than the women with high income, [AOR = 3.97, 95% CI: 1.81–8.72]. Women whose bride price as requested by her parent was partially paid or not paid at all were 3.46 times more likely to experience IPV, than women where the requested bride price had been paid, [AOR = 3.46; 95% CI: 1.74–6.87]. Women who indicated that a married women has no right to refuse to have sex with her husband in some situations such as sickness etc. were thrice as likely to experience IPV as women who believed a woman could refuse sex in some situations, [AOR = 2.99; 95% CI:1.39–6.41]. Women whose partner drank alcohol were 2.36 times more likely to experience IPV than those whose partner did not drink alcohol, [AOR = 2.36; 95% CI: 1.36–4.10]. Similarly, women who had between two and nine sexual partners were 2.35 times more likely to experience IPV than women, who had one sexual partner, [AOR = 2.35; 95% CI: 1.36–4.09]. Women, whose partner had ever been involved in a physical fight with another man, were 1.83 times more likely to experience IPV than those women who did not report this, [AOR = 1.83; 95% CI: 1.05–3.19].

Women who had experienced their behaviour being controlled by their husband were 8.13 times more likely to suffer IPV than those women, who did not report this, [AOR = 8.13; 95% CI: 4.93–13.42]. Women whose partner’s family arranged their marriage were 2.82 times more likely to experience IPV, compared to those couples who chose marriage together [AOR = 2.82; 95% CI:1.70–4.69] (Table 5).

**Table 5:** Logistic regression model of factors associated the lifetime IPV against women living with and without HIV in Ethiopia, November-December 2018.

Characteristics (n = 816)	Lifetime physical, sexual and emotional violence (IPV)		COR(95%CI)	AOR(95%CI)	P-value
	Yes	No			
**Age group of women**					
18–28	141(45.63)	168(54.37)	1.00	1.00	
29–39	287(66.59)	144(33.41)	2.37(1.76–3.21)	2.86(1.67–4.90)*	**p<0.001**
40–49	59(77.63)	17(22.37)	4.14(2.31–7.42)	1.86(0.70–4.93)	P = 0.211
**Wealth Index**					
Poor	207(73.14)	76(26.86)	2.75(1.91–3.94)	3.97(1.81–8.72)*	**P = 0.001**
Middle	154(55)	126(45)	1.23(0.88–1.73)	1.46(0.82–2.59)	P = 0.199
High	126(49.68)	127(50.20)	1.00	1.00	
**Women’s Education**					
No Education	128(69.95)	55(30.05)	4.03(2.26–7.17)	0.63(0.20–1.98)	P = 0.431
Primary Education (1–8)	206(61.86)	127(38.14)	2.81(1.65–4.77)	0.72(0.28–1.86)	P = 0.503
Secondary (9–12 Grade)	127(55.62)	102(44.54)	2.15(1.24–3.73)	0.96(0.39–2.40)	P = 0.943
Higher (Above 12)	26(36.62)	45(63.38)	1.00	1.00	
**Payment of bride price**					
All paid	267(54.71)	221(45.29)	1.00	1.00	
Partially paid or Non paid	81(72.97)	30(27.03)	2.23(1.42(3.52))	3.46(1.74–6.87)*	**p<0.001**
**Attitude to refuse to have sex in some situations**					
Believe no right to refuse	121(76.58)	37(23.42)	2.61(1.75–3.89)	2.99(1.39–6.41)*	**P = 0.005**
Can refuse sex in some situations	366(55.62)	292(44.38)	1.00	1.00	
**Partner consumed alcohol**					
Yes	243(75.23)	80(24.77)	3.10(2.28–4.22)	2.36(1.36–4.10)*	**P = 0.002**
No	244(49.49)	249(50.51)	1.00	1.00	
**Number of sexual partner**					
One(1)	284(53.28)	249(46.72)	1.00	1.00	
Two-nine(2–9)	203(71.73)	80(28.27)	2.22(1.63–3.03)	2.35(1.36–4.09)*	**P = 0.002**
Partner ever involved in a physical fight with another man					
Yes	203(76.32)	63(23.68)	3.45(2.47–4.82)	1.83(1.05–3.19)*	**P = 0.034**
No	229(48.31)	245(51.69)	1.00	1.00	
Do not Know	55(72.37)	21(27.63)	2.80(1.64–4.78)	1.61(0.65–3.40)	P = 0.306
**Controlled behaviour of husband (Composite score)**					
Yes	389(79.55)	100(20.45)	9.09(6.58–12.55)	8.13(4.93–13.42)*	**p<0.001**
No	98(29.97)	229(70.03)	1.00	1.00	
**Who chose marriage**					
Both chose	199(49.63)	202(50.37)	1.00	1.00	
Respondent chose	17(58.62)	12(41.38)	1.44(0.67–3.09)	1.57(0.49–5.06)	P = 0.452
Respondent’s family chose	19(70.37)	8(18.18)	2.41(1.03–5.64)	1.34(0.27–6.74)	P = 0.716
Partner chose	9(81.82)	2(18.18)	4.57(0.97–21.41)	2.94(0.21–41.82)	P = 0.427
Partner’s family chose	239(70.71)	99(29.29)	2.45(1.81–3.33)	2.82(1.70–4.69)*	**p<0.001**
Others	4(40)	6(60)	0.68(0.19–2.43)	0.55(.01–49.53)	0.794

* = Statistically significant in multivariable logistic regression (AOR)

## Discussion

This study assessed the prevalence of IPV among women living with and without HIV and the associated factors, and demonstrates a significant burden of IPV among women, amongst both groups in Wolaita Zone, Ethiopia. The study found that the overall lifetime IPV among all women surveyed was 487 (59.68%), [95% CI: 56.31%-63.05%], whereas ten previous studies reported IPV prevalence from 20 to 78% [16]. This study from Southern Ethiopia confirms the high prevalence of IPV in this region. In Ethiopia Dhairyawan et al. (2013) reported lifetime IPV prevalence of 52% [17], and Yigzaw et al. in Northern Ethiopia in 2004 found an IPV prevalence of 50.8% [18]. These statistics are higher than the WHO African, Eastern Mediterranean, and South-East Asian regions study which reported a lower IPV prevalence of 37% [3]. A possible explanation for these results may be the lack of adequate awareness about women’s reproductive health rights among the current study population and also differences in the study setting.

The current study found no significant differences in lifetime prevalence of IPV among women living with HIV, 250(61.3%), [95% CI: 56.53%-66.02%] compared to women who were HIV negative, 238(58.1%), 95% CI: 53.28%-62.90%] (p = 0.354). This finding is in agreement with the study conducted in ten developing countries by Harling, et al., (2010), in which there was no association found between HIV infection and IPV[19].

The lifetime prevalence of each of the factors, emotional, physical, and sexual violence among all women interviewed (n = 816) was 413 (50.61%), 349(42.77%), and 219 (26.84%), respectively, indicating that emotional violence was the most common form of violence. The present findings appear to be consistent with other research which found that the mean lifetime prevalence of domestic emotional violence was 51.7% [8]. However, the findings of the current study were lower than a study conducted in North West Ethiopia, in which the psychological, physical, and sexual violence were 73.3%, 58.4% and 49.1%, respectively [16]. A possible explanation for this result may be that the North West Ethiopia study was conducted only in a rural setting, with a small sample size and cultural differences.

The prevalence of IPV among all women in the last 12 months preceding the survey was 78 (33.91%) (n = 230).This finding is in agreement with the WHO global study, where the prevalence was 30%, and for the study in India, 35% [20], and also 35% for the Ethiopian DHS 2016 study [9]. However, a prevalence of IPV (21%) was found in a study in Cape Town, Southern African, among 623 HIV–infected pregnant women in 2016 [21]. A possible explanation could be that in the Cape Town study, the participants were only from urban areas, where the women may have been more empowered and they may have different awareness levels to the women in this study.

The reported physical and sexual violence in the last 12 months among all the women in this study was, 30.86% and 32.39% respectively. The present findings seem to be consistent with other research which found similar prevalence of physical and sexual violence in the past year (28% for each) [14]. However, the findings of the current study are higher than that of the South African study in which the reported physical and sexual violence was 15% and 2%, respectively [21], and the Ethiopian DHS study, in which the prevalence of physical and sexual violence in the past 12 months preceding the survey was 15% and 7%, respectively [9]. A possible explanation for this might be differences in the study areas, and the beliefs and culture of the societies. This finding emphasizes that IPV is of public health concern in Ethiopia. The magnitude of the problem is not just from a human rights’ perspective, but also because of the negative economic and health aspects affecting women in Ethiopia [12].

The results of this study did not show any significant association between the HIV status of the women (COR = 1.14; 95% CI: 0.86–1.51), nor their own or their husbands’/partners’ education level or occupation, residence, or religion. These findings seem to be consistent with studies of South West Ethiopia and other research in ten developing countries [14, 19]. Contrary to this finding, the residence, occupation and educational status of women were significantly associated with IPV in the study conducted in Ethiopia among women of reproductive age [8]. Such differences may be related to the difference in the study setting among the Ethiopian populations and their different cultures. The lower income and older age group of women (29–39 years) were significantly associated with IPV among the socio-demographic variables. These results are similar to those observed in earlier studies concerning the association of women with lower income with IPV [16]. Income is a key factor to assist women to protect themselves from violence. If women are less dependent on their partner and empowered economically, they are less likely to be abused, and hence women need to be assisted to engage in income generating activities [22–24]. When women start to earn their own income, they are less dependent upon their male partners, and this provides less opportunity for male partners to control their wives and the men may lose some of their power [22].

In contrast to this finding, a study conducted in the UK showed that the older age group of women was not significantly associated with IPV [17]. It could perhaps be explained by the differences in the study setting and economic independence of women in UK compared to Ethiopia.

The current study found that women whose husband/partner controls their wives’ behaviour were more likely to experience IPV than women who did not experience such control over their behaviour. This finding is consistent with the studies conducted by Deribe et al. (2012) and Shamu et al.2014 [14, 25]. Such controlling tactics are a risk for women’s physical and mental health promoting pain / discomfort, sadness or depression, and perhaps even suicidal ideation (26). Controlling behaviours may restrict women from seeking medical care and contact from family and friends. Their partners’ suspicions that women are unfaithful are also part of such controlling behaviours[26].

This study found that having a male partner who drank alcohol was significantly associated with IPV. This finding is in agreement with the previous research done by Semahegn (2015) and Yigzaw (2004) [16, 18]. A possible explanation for these results may be that a person who consumed alcohol might be likely to be aggressive and angry and to abuse women emotionally, physically and /or sexually. Different studies have shown that alcohol leads to IPV and sexual risk taking, such as engagement with commercial sex workers and reduced condom use [27, 28].

An interesting finding regarding the prevalence of violence was that the women who reported risky sexual behaviour, particularly having least two or more partners, and those whose partners had been involved in a physical fight with another man were at increased risk of IPV. These findings of the current study are consistent with those of Shamu et al. (2014) [25] who found that if a partner ever fought with another man this was statistically associated with IPV. Women who have multiple sexual relations with several partners appear to be at increased risk of experiencing abusive behaviour from their partners.

Regarding the decision making powers of a woman, those women who reported that they were unable to refuse sex in some situations, were at significant risk of being abused by their partner. This was associated with IPV and suggests these women’s powerlessness placed them at risk (P = 0.005). This finding is in agreement with the study by Shamu et al. 2014 [25] who explained that women endorsing three to six sexual abuse attitudes, were more likely to experience IPV than those women who were less likely to have positive attitudes to abuse.

Women, whose bride price payment was only partially paid or not paid at all, were more likely to have experienced IPV than their counterparts. This is also in agreement with the finding of the study conducted in Western Ethiopia, 2016 [29]. This may occur since the cultural obligations have not been met and as marriages in Ethiopia are often arranged by the parents of the bride and groom, these require much negotiation. At the time of the wedding ceremony, the bride’s parents give the groom a dowry. When the expected bride-price was not paid, the family or the groom might act violently. Women whose partner’s family arranged their marriage were statistically more likely to experience IPV. In a marriage arranged by their parents, there might be difficulties as the couple had not known each other before such arrangement[30]. In summary, IPV is highly prevalent in southern Ethiopia and mainly highly associated with male controlling behaviour, their alcohol drinking and low income of women. Therefore, changing male attitudes through awareness creation and involving them in IPV prevention programs are important. Moreover, the economic empowerment of women is a very crucial intervention (24).

## Strength and limitations

The present study provides additional evidence with respect to IPV among women living with and without HIV and reports a high prevalence of IPV in southern Ethiopia. The strengths of this study are that it included the special population of women who are living with HIV. It also gave an opportunity for women living with and without HIV to share and explain their experience of violence to others and to contribute to the recommendations provided by this study. The study benefitted from the use of a large stratified random sample and the use of the standard WHO questionnaire. On the other hand, the generalizability of these results is subject to certain limitations as it is an institutionally based study. Women who have not tested for HIV and women who are HIV positive but not taking ART were not included when selecting the study sample. Further, a cross-sectional study cannot establish the temporal relationship, though the design helps to examine the association with a number of variables. The cross-sectional study thus cannot support conclusions regarding the inference on causality or on the risk of diseases or in this study, IPV.

## Conclusion

These results indicate that there is a high prevalence of IPV against women in Wolaita Zone, both those living with and without HIV. Emotional violence was the most common form of IPV followed by physical and sexual violence. These results suggest an association between IPV and the age group of women (older women are at increased lifetime risk). Women’s lack of power is indicated by the association of IPV and income, bride price payment, and women’s attitude to refuse to have sex. IPV was also associated with alcohol consumption by the husband/partner, number of sexual partners, whether husband/partner ever physically fought with another man, the controlling behaviour of the husband/ partner, and the husband’s family arranging their marriage. In order to tackle IPV there is a need to empower women to make their own decisions about their reproductive health. A key policy priority should be to involve males in programs of gender-based violence prevention in order to change their violent behaviour. Additionally, improving the economic status of women, advising women /men to limit the number of sexual partners and that marriage arrangements should be by mutual consent rather than made by parents, are also recommended. Further investigations are needed to monitor the physical and mental health impact of IPV among different groups of women and different regions.

## Supporting information

S1 File English questionnaire. (PDF)

S2 File Amharic questionnaire. (PDF)

S3 File Wolaita language version. (PDF)

S4 File Ethical Approval. (PDF)

S5 File IRB Approved WSU Ethiopia. (PDF)
