# Supplementary material for: Intimate partner violence against women living with and without HIV, and the associated factors in Wolaita Zone, Southern Ethiopia: A comparative cross-sectional study
Source: PLoS One. 2019 Aug 23;14(8):e0220919. doi: 10.1371/journal.pone.0220919 (PMC6707594; doi:10.1371/journal.pone.0220919)
Supplement: S1 File — (PDF) [file pone.0220919.s001.pdf]

Quantitative study Information sheet, consent and questionnaire for women

Information Sheet and Consent to Participate in Research

Date: --------------------------------
How are you? My name is ---------------------------- I provide the information about the researcher Mr. Mengistu Meskele Koyira, a Ph.D student at university of KuwaZulu -Natal, and former staff of Wolaita Sodo university, his contact address is (+251913177996 or E-mail address: mengistu77@gmail.com). You are being invited to consider participating in a study that involves research in **"Intimate partner violence against women living with and without HIV, and the associated factors in Wolaita Zone, Southern, Ethiopia: A comparative cross-sectional study"**. The aim and purpose of this research is to learn about the women's health and life experiences and to generate the local evidence. The study is expected to enroll 816 participants. We will conduct this study in the nine health facilities in Wolaita Zone. It will involve the following procedures. We are going to ask you questions on women's health and life experience. All answers that you will provide me are acceptable. The duration of your participation if you choose to enroll and remain in the study is expected to be 45 minutes.
The study may involve the following risks and/or discomforts. By participating in this research project, you may have minimum discomfort to disclose your painful experience, though; many women have found it helpful to have the opportunity to talk. We will also refer to psychological intervention or counseling service at health care facility in case you get discomfort. We (the research assistant) will also provide counseling at the end of the interview. The study helps to generate local evidence to assist with the initiation and implementation of programs to address intimate partner violence against women.

This study has been ethically reviewed and approved by the UKZN Biomedical research Ethics Committee (approval number---------------------------------).

In the event of any problems or concerns/questions you may contact the researcher Mr.Mengistu Meskele (at +251913177996 or E-mail address: mengistu77@gmail.com) or the UKZN Biomedical Research Ethics Committee, contact details as follows:

Biomedical Research Ethics Administration

1. Research Office, Westville Campus
2. Govan Mbeki Building

Private Bag X 54001

Durban

4000

KwaZulu-Natal, SOUTH AFRICA

Tel: 27 31 2604769 - Fax: 27 31 2604609

Email: BREC@ukzn.ac.za

Your participation in this research is completely voluntary. Your experiences could be very helpful to other women in Ethiopia. You may withdraw participation at any point, and that in the event of refusal/withdrawal of participation; the participants will not incur penalty or loss of treatment or other benefit to which they are normally entitled.

There is no direct benefit to your participation, but many women have found it helpful to have the opportunity to talk.

I kindly let you know that all of the answers you provide me will be kept secret. The record of your name or address will not be kept by the researcher. Any information from the participants like raw data will be stored securely and kept confidential. The publications that will arise from this study will exclude any information that will make it possible to identify the participants' name or identity. In case others enter the room, dummy questionnaires will be used.

CONSENT

I (---------------------------------) have been informed about the study entitled (**Intimate Partner Violence Against Women living with and without HIV: Contexts and associated factors in Wolaita Zone, Ethiopia**) by (Mr. Mengistu Meskele Koyira).

I understand the purpose and procedures of the study.

I have been given an opportunity to answer questions about the study and have had answers to my satisfaction.

I declare that my participation in this study is entirely voluntary and that I may withdraw at any time without affecting any treatment or care that I would usually be entitled to.

I have been informed about any available compensation or medical treatment if injury occurs to me as a result of study-related procedures. If I have any further questions/concerns or queries related to the study I understand that I may contact the researcher at (Mr. Mengistu Meskele (+251913177996 or E-mail address: mengistu77@gmail.com)

If I have any questions or concerns about my rights as a study participant, or if I am concerned about an aspect of the study or the researchers then I may contact:

Biomedical Research Ethics Administration

1. Research Office, Westville Campus
2. Govan Mbeki Building

Private Bag X 54001
Durban
4000
KwaZulu-Natal, SOUTH AFRICA
Tel: 27 31 2604769 - Fax: 27 31 2604609
Email: BREC@ukzn.ac.za

Signature of Participant Date

______________________ _______________________

Witness name

1.---------------------------------- ------------------------------------

2.---------------------------------- ------------------------------------

3. Interviewer name --

Questionnaire IV: Quantitative Study

Section 1. Socio-demographic and economic characteristics

No	Questions	Response	Skip
	Client HIV status	1. ART client 2. HIV negative women	
S1	How old are you now?	Age in completed years-----------------------	
S2	What is your residence type?	1.Urban 2. Rural	
S3	What is your current Marital status?	1. Currently married/Living with man →	GoS7
		2. Living with man not married →	GoS7
		3. Currently having a regular partner living apart →	GoS7
		4. Not currently married or living with a man	
S4	Have you ever been married or lived with a male partner?	1. Yes, married<br>2. Yes, lived with a man, but never married<br>3. . No	
S5	Did the last partnership with a man end in divorce or separation, or did your husband /partner die?	Divorced ....1<br>Separated/broken up .... 2<br>Widowed/partner died ....3 →	Go S7
S6	Was the divorce/separation initiated by you, by your husband/partner, or did you both decide that you should separate?	Respondent......................1<br>Husband/partner ....2<br>Both (respondent and partner) ....3<br>Other: ________________.....4	

S7	How many times in your life have you been married and/or lived together with a man?	Number of times married/ lived together ------<br>Refused/no answer ----------------------8	
S8	Does/did your husband/partner have any other wives while being married (having a relationship) with you?	Yes..1<br>No..**2**<br>Don't know/don't remember.....**3**<br>Refused/no answer 4	
S9	How many wives/partners does/did he have (including yourself)?	Number of wives ....<br>Refused/no answer 8	
S10	Are/were you the first,second.....wife/partner?	Number /position .... [ ][ ]<br>Refused/no answer 8	
S11	Did you yourself choose your current/most recent husband, did someone else choose him for you, or did he choose you?<br>IF SHE DID NOT CHOOSE HERSELF, PROBE:<br>Who chose your current/most recent husband/partner for you?	Both chose ....**1**<br>Respondent chose..........................**2**<br>Respondent's family chose ....3<br>Partner chose ....4<br>Partner's family chose................... 5<br>Church...6<br>Don't know/don't remember .. ……7	
S12	Before the marriage with your current /most recent husband, were you asked whether you wanted to marry him or not?	YES ....1<br>NO...2<br>DON'T KNOW/DON'T REMEMBER ...3<br>REFUSED/NO ANSWER 4	
S13	Did your marriage involve bride price payment?	Yes /dowry ....1<br>Yes/ bride price.....................................2<br>No ....**3**<br>DON'T KNOW/DON'T REMEMBER ..**4**	
S14	Has all of the bride price been paid for, or does some part still remain to be paid?	1.All paid 2 .Partially paid<br>3 .None paid<br>4 .Don't know/don't remember	
S15	What is the size of your house hold?	-----------------------------------	
S16	What is your religion?	1.Orthodox 2.Muslim 3.Protestant<br>4. Catholic 5. Traditional<br>6.Apostolic 7. Other, specify	
S17	What is your ethnicity?	1.Wolaita 4. Gurage	

		2.Amara 5. Oromo<br>3. Dawuro 6. Others/specify---	
S18	What is your occupation?<br>(Women)?	1. House wife 2. Trader 3. Student<br>4. Government/NGO employee 5. Daily laborer<br>6. others, specify-----------------------------	
S19	What is your husband's occupation?	1. Farmer 2. Government employ 3. Merchant 4. Daily laborer 5. NGO 6. Other	
S20	Have you ever attended formal school?	1. Yes<br>2. No	If no go S22
S21	What is the highest grade you have completed?	Grade completed.......................................	
S22	Has your husband attended formal education?	Yes…………………………1<br>No…………………………2	"no" GO S24
S23	What is your husband's highest grade he has completed?	Formal grade completed---------------------	
S24	What is your monthly income?	Ethiopian Birr: ---------------------------------	
S25	Does your household have a functioning ………………: [write '1' if yes & '0' if no.]<br>W1. Television: |___| W2. Radio: |___| W3. Satellite dish: |___| W4. Mobile phone |___| W5. Cell phone (home)|___| W6. Table |___| W7. Chair: [ ] 8.Bed/cotton/spring/matters/ 9.Electric Mitad |___| W10. Bicycle: |___| W11. Motor bicycle /Bajaji |___| W12. Refrigerator |___] W13. Electricity 14. Tractor/Car 15. Farm land/town land 16. 'Enjera Mitad' (Wood) 17. Electric stove [ ]		

Section II. Thinking about your (current or recent) husband / partner, would you say it is generally true that he **(Decision making subscale)**

S.No	Questioner	Yes	No	DK
D27	Tries to keep you from seeing your friends?	1	2	8
D28	Tries to restrict contact with your family of birth?	1	2	8
D29	Insists on knowing where you are at all times?	1	2	8
D30	Ignores you and treats you indifferently?	1	2	8
D31	Gets angry if you speak with another man?	1	2	8
D32	Is often suspicious that you are unfaithful?	1	2	8
D33	Expects you to ask his permission before seeking health care for yourself?	1	2	8

Section III: The next questions are about things that happen to many women, and that your **current partner, or any other partner** may have done to you. Has your current husband or partner any other partner ever? **(Emotional Violence)**

S.N.	Questions	A) If Yes continue with B. If NO skip to next Item		B) Has this happened in the past 12 months? (If YES ask C only. If NO ask D only)		C)In the past 12 months would you say that this has happened once, a few times or many times? (after answering C, go to next item)			D)Before the past 12 months would you say that this has happened once, a few times or many times?		
		Yes	No	Yes	No	One	Few	Many	One	Few	Many
E34	Insulted you or made you feel bad about yourself?	1	2	1	2	1	2	3	1	2	3
E35	Belittled or humiliated you in front of other people?	1	2	1	2	1	2	3	1	2	3
E36	Done things to scare or intimidate you on purpose (e.g. by the way he looked at you, by yelling and smashing things)?	1	2	1	2	1	2	3	1	2	3
E37	Threatened to hurt you or someone you care about?	1	2	1	2	1	2	3	1	2	3

S.N.	Questions **(Physical Violence)** **Has he or any other partner ever….**	A) If YES continue with B. If NO skip to next Item		B) Has this happened in the past 12 months? (If YES ask C only. If NO ask D only)		C)In the past 12 months would you say that this has happened once, a few times or many times? (after answering C, go to next item)			D)Before the past 12 months would you say that this has happened once, a few times or many times?		
		Yes	No	Yes	No	One	Few	Many	One	Few	Many
P38	Slapped you or thrown something at you that could hurt you?	1	2	1	2	1	2	3	1	2	3
P39	Pushed you or shoved you or pulled your hair?	1	2	1	2	1	2	3	1	2	3
P40	Hit you with his fist or with something else that could hurt you?	1	2	1	2	1	2	3	1	2	3
P41	Kicked you, dragged you or beat you up?	1	2	1	2	1	2	3	1	2	3
P42	Choked or burnt you on purpose?	1	2	1	2	1	2	3	1	2	3
P43	Threatened to use or actually used a gun, knife or other weapon against you?	1	2	1	2	1	2	3	1	2	3

S.N.	**(Sexual Violence)** **Has he or any other partner ever….**	A) If YES continue with B. If NO skip to next Item		B) Has this happened in the past 12 months? (If YES ask C only. If NO ask D only)		C)In the past 12 months would you say that this has happened once, a few times or many times? (after answering C, go to next item)			D)Before the past 12 months would you say that this has happened once, a few times or many times?		
		Yes	No	Yes	No	One	Few	Many	One	Few	Many
S44	Did your current husband/partner or any other partner ever physically force you to have sexual intercourse when you did not want to?	1	2	1	2	1	2	3	1	2	3
S45	Did you ever have sexual intercourse you did not want to because you were afraid of what your partner or any other partner might do?	1	2	1	2	1	2	3	1	2	3
S46	Did your partner or any other partner ever forced you to do something sexual that you found degrading or humiliating?	1	2	1	2	1	2	3	1	2	3

S.N.				
P47	Verify whether answered yes to any Question on physical violence? See Question Number P38-S43.	1. Yes, physical violence<br>2. No physical violence		
S48	Verify whether answered yes to any question on sexual violence, See question S44-S46	1. Yes, sexual violence<br>2. No sexual violence		
I49	Have ever been pregnant?	1.Yes<br>2.No If 2 Go to I52		
I50	You said that you have been pregnant TOTAL times. Was there ever a time when you were slapped, hit or beaten by (any of) your partner(s) whilst you were pregnant?	1. Yes<br>2. No<br>3. Don't know/don't remember If 2,3,4 Go to I53<br>4. Refused/no answer		
I51	Did you miscarry as a result of violence?	1. Ye<br>2. No		
I52	Have you ever been injured (physical harm, including cuts, sprains, burns, broken bones or broken teeth) as a result of these acts by (any of) your husband / partner(s). Please think of the acts that we talked about before.		Yes	No
		1. cuts, punctures, bites	1	2
		2. scratches, abrasion, bruise	1	2
		3. Sprains, dislocations	1	2
		4. Burns	1	2
		5. Penetrating injury, deep cuts, gashes?	1	2
		6. Broken eardrum, eye injuries	1	2
		7. Fractures, broken bones	1	2
		8. Broken teeth	1	2
		9. Others	1	2
I53	In your life, how many times were you injured by (any of) your husband/partner(s)?<br>Would you say once or twice, several times or many times?	1. Once/twice<br>2. Several (3-5) times<br>3. Many (more than 5) times<br>4. Don't know / don't remember<br>5. Refused / no answer		
I54	Has this happened in the past 12 months?	1. Yes<br>2. No<br>3. Don't know / don't remember		

		4. Refused / no answer
I55	Did you receive health care for your injury?	1. Yes some times<br>2. Yes always<br>3. No
I56	Have you ever tried to seek help?	1.Yes 2. No
I57	Who did you tell about this behavior of your partner (physical violence)?	NO ONE ....1<br>FRIENDS...2<br>PARENTS..3<br>BROTHER OR SISTER.. ..4<br>UNCLE OR AUNT ....5<br>HUSBAND/PARTNER'S FAMILY ....6<br>CHILDREN ....7<br>NEIGHBOURS... 8<br>POLICE ....9<br>DOCTOR/HEALTH WORKER..................................10<br>Religious leader.. ..11<br>COUNSELLOR ....12<br>NGO/WOMEN'S ORGANIZATION ....13<br>LOCAL LEADER .... 14<br>LAW ORGANISATION..15<br>OTHER (specify): ____________________________16
I58	Did anyone try to help you?<br>Who tried to help you?<br>MARK ALL MENTIONED<br>PROBE: Who else?	NO ONE ....1<br>FRIENDS...2<br>PARENTS..3<br>BROTHER OR SISTER.. ..4<br>UNCLE OR AUNT ....5<br>HUSBAND/PARTNER'S FAMILY ....6<br>CHILDREN ....7<br>NEIGHBOURS... 8<br>POLICE ....9<br>DOCTOR/HEALTH WORKER..................................10<br>Religious leader.. ..11<br>COUNSELLOR ....12<br>NGO/WOMEN'S ORGANIZATION ....13<br>LOCAL LEADER .... 14<br>LAW ORGANISATION..15<br>OTHER (specify): ___________________________16

W59	During the times that you were hit, did you ever fight back physically or to defend yourself?<br>**1.Yes**<br>**2.No**<br>IF YES: How often? Would you say once or twice, several times or most of the time?	1. Once or twice<br>2. Many times, /most of the time<br>3. Don't know / don't remember<br>4. Refused / no answer
W60	Have you ever hit or physically mistreated your husband/partner when he was not hitting or physically mistreating you?<br>1.Yes 2. No , IF YES: How often? Would you say once or twice, several times or many times?	1. Once or twice<br>2. Many times<br>3. Don't know / don't remember<br>4. Refused / no answer

Section: Life time experience of Violence from relatives, other people that they know, and/or from strangers.

S.No.	Question		Ask only for those marked. How many times did this happen?		
			Once or Twice	A few times	Many times
L61	**<u>Since the age of 15</u>**, has anyone (FOR WOMEN WITH CURRENT OR PAST PARTNER: other than your partner/husband) ever beaten or physically mistreated you in any way?<br>1.Yes<br>2. No, IF YES: Who did this to you?<br>PROBE:<br>How about a relative?<br>How about someone at school or work?<br>How about a friend or Neighbor?<br>A stranger or anyone else?	1.none	1	2	3
		2. Father	1	2	3
		3. Step father	1	2	3
		4. Other male family member	1	2	3
		5. Female family member	1	2	3
		6. Teacher	1	2	3
		7. Police/ soldier	1	2	3
		8. Male friend of family	1	2	3
		9. Female friend of family	1	2	3
		10. Boyfriend	1	2	3
		11. Stranger	1	2	3
		12. Someone at work	1	2	3
		13. Priest/religious	1	2	3
		14. Leader	1	2	3

S. No.	Question		How old were you during such incident?	How old were this person during such incident?	Ask only for those marked. How many times did this happen?		
					Once or Twice	A few times	Many times
L62	**Before the age of 15**, do you remember if anyone in your family ever touched you sexually, or made you do something sexual that you didn't want to?<br>1.Yes<br>2.No<br>IF YES: Who did this to you? IF YES OR NO CONTINUE:<br>How about someone at school?<br>How about a friend or neighbor?<br>Has anyone else done this to you?<br>IF YES: Who did this to you?	1.none			1	2	3
		2. Father			1	2	3
		3. Step father			1	2	3
		4. Other male family member			1	2	3
		5. Female family member			1	2	3
		6. Teacher			1	2	3
		7. Police/ soldier			1	2	3
		8. Male friend of family			1	2	3
		9. Female friend of family			1	2	3
		10. Boyfriend			1	2	3
		11. Stranger			1	2	3
		12. Someone at work			1	2	3
		13. Priest/religious			1	2	3
		14. Leader			1	2	3

S.N.	**Characteristics**	Response	Skip
L63	What did you do your relationship when you have encountered physical and or sexual violence?	1. Leaves relationship temporarily<br>2. Leaves relationship Permanently<br>3. I didn't encounter violence<br>4. Other	
L64	What are the consequences of violence in your life?	1. Non-applicable<br>2. Self-humiliation<br>3. Problems in health and treatment<br>4. Social discrimination<br>5. Low access to basic needs	
H65	Does /Did your partner drinking alcohol?	1. Yes<br>2. No	If 2 L69
H66	How often does(did) he gets drunk? Often, only sometimes, or never?	1. Every day or nearly every day<br>2. Once or twice a week<br>3. 1 – 3 times in a month<br>4. Rarely<br>5. Don't know/don't remember<br>6. Refused	

S.N	Questions	Response			Response
H68	In the past 12 months, have you ever experienced any of the following problems, related to HIS drinking?<br>a) money problems<br>b) health problems<br>c) conflict with family or friends<br>d) problems with authorities (bar owner/police, etc)<br>x) other, specify		Yes	No	
		A) Money problems	1	2	
		B) Health problems	1	2	
		C) Conflict with family Or friends	1	2	
		D) Problems with Authorities	1	2	
		E) Other:	1	2	
L69	Just now we talked about problems that may have bothered you in the past 4 weeks. I would like to ask you now: In your life, have you ever thought about ending your life?	Yes ....1<br>No................2<br>Don't know/don't remember ....3<br>Refused/no answer 4			
L70	Have you ever tried to take your life?	YES ....1<br>NO 2			

		DON'T<br>KNOW/DON'T REMEMBER.........3<br>REFUSED/NO ANSWER……………….4	
H71	Have you ever asked your current/most recent partner to use a condom?	YES...1<br>NO ....2<br>Don't know/Don't remember......................3<br>REFUSED..4	
H72	Has your current/most recent husband/partner ever refused to use a condom?	YES.. 1<br>NO .... 2<br>Don't know/Don't remember.........................3<br>REFUSED...4	If 1 Go L74
H73	In what ways did he let you know that he disapproved of using a condom?<br>MARK ALL THAT APPLY	TOLD ME HE DID NOT APPROVE .... A<br>SHOUTED/GOT ANGRY .... B<br>THREATENED TO BEAT ME .... C<br>THREATENED TO LEAVE/THROW ME<br>OUT OF HOME .... D<br>BEAT ME/PHYSICALLY ASSAULTED.......E<br>TOOK OR DESTROYED METHOD ....F<br>ACCUSED ME OF BEING UNFAITHFUL/<br>NOT A GOOD WOMAN......................... G<br>LAUGHED AT/NOT TAKE ME SERIOUS .. H<br>SAID IT IS NOT NECESSARY ....I<br>OTHER ________________________________ . X	
L74	Now I would like to ask you about your first sexual intercourse. At what age were you when you first had sexual intercourse?	Completed year----------------------------	
L75	Which of the following statements most closely describes your experiences the first time you had sexual<br>intercourse?<br>I was willing; I was persuaded; I was tricked; I was forced; I was raped	I was willing..............................1<br>I was persuaded........................2<br>I was tricked.............................3<br>I was forced ....4<br>I was raped................................5	
L76	Who was this with?	Husband/partner........................1<br>Boyfriend...................................2<br>Teacher......................................3<br>Father/family member................4<br>Man from school/area................5<br>Friend of the family.....................6<br>Relative.......................................7<br>stranger/unknown person...........8<br>Others....................................... 9	
L77	How old was he when you had sexual intercourse with him? Would you say he was.......	Year [ ][ ]<br>Younger than me.........................1<br>Same age with me.........................2<br>1-2 years older than me……………..3<br>3-5 years older than me……………..4	

		5-10 years older than me……………5<br>More than 10 years older than me...6<br>REFUSED/NO ANSWER …………………9	
L78	The number of sexual partners women have had differs a lot from person to person. Some women report having<br>had one sex partner, some 2 or more, and still others report many, even 50 or more. In your life how many different men have you had sex with?<br>IF NEEDED PROBE: More or less; I do not need to know the exact number.	PARTNERS .... [ ][ ][ ]<br>DON'T KNOW/DON'T REMEMBER......................1<br>REFUSED/NO ANSWER......................................2	
L79	When you were a child, was your mother hit by your father (or her husband or boyfriend)?	YES...1<br>NO...2<br>PARENTS DID NOT LIVE TOGETHER......................3<br>DON'T KNOW..4<br>REFUSED/NO ANSWER 5	
L80	As a child, did you see or hear this violence?	YES..1<br>NO...2<br>DON'T KNOW.......................................3<br>REFUSED/NO ANSWE...........................4	
H81	As far as you know, was your (most recent) partner's mother hit or beaten by her husband?	YES..1<br>NO ....2<br>PARENTS DID NOT LIVE TOGETHER ....3<br>DON'T KNOW ....4<br>REFUSED/NO ANSWER 5	
H82	Did your (most recent) husband/partner see or hear this violence?	YES..1<br>NO...2<br>DON'T KNOW.................................3<br>REFUSED/NO ANSWER 4	
H83	As far as you know was your current/most recent husband/partner beaten regularly by someone in his family?	YES...1<br>NO..2<br>DON'T KNOW/DON'T REMEMBER....3<br>REFUSED/NO ANSWER.......................4	
H84	Since you have known him, has he ever been involved in a physical fight with another man?	YES ....1<br>NO ....2<br>DON'T KNOW /DON'T<br>REMEMBER…………….3<br>REFUSED/NO ANSWER 4	
	SECTION: **ATTITUDES TOWARDS PARTNER BEATING**<br><br>In this community and elsewhere, people have different ideas about families and what is acceptable behaviour for men and women in the home. I am going to read to you a list of statements, and I would like you to tell me whether you generally agree or disagree with the statement. There are no right or wrong answers		

A85	In your opinion, does a man have a good reason to hit/beat his wife if:<br>a) She does not complete her household work to his satisfaction<br>b) She disobeys him<br>c) She refuses to have sexual relations with him<br>d) She asks him whether he has other girlfriends<br>e) He suspects that she is unfaithful<br>f) He finds out that she has been unfaithful	 <br><br>A) HOUSEHOLD<br>B) DISOBEYS<br>C) NO SEX<br>D) GIRLFRIENDS<br>E) SUSPECTS<br>F) UNFAITHFUL	Yes<br><br><br>1<br>1<br>1<br>1<br>1<br>1	No<br><br><br>2<br>2<br>2<br>2<br>2<br>2	DK<br><br><br>3<br>3<br>3<br>3<br>3<br>3	
A86	In your opinion, can a married woman refuse to have sex with her husband if:<br>a) She doesn't want to<br>b) He is drunk<br>c) She is sick<br>d) He mistreats her<br>e) She suspects he has extra-marital sexual relations with another woman<br>f) She suspects her husband has an STI/HIV	A) NOT WANT<br>B) DRUNK<br>C) SICK<br>D) MISTREAT<br>E) SUSPECTS EXTRA MARITAL SEX<br>F) SUSPECTS/KNOW STI	1<br>1<br>1<br>1<br>1<br>1<br><br>1	2<br>2<br>2<br>2<br>2<br>2<br><br>2	3<br>3<br>3<br>3<br>3<br>3<br><br>3	
	RESPONDENT AND HER PARTNER					
H87	In general, do (did) you and your (current or most recent) husband/partner discuss the following topics together:<br>a) Things that have happened to him in the day<br>b) Things that happen to you during the day<br>c) Your worries or feelings<br>d) His worries or feelings	 <br>a) HIS DAY<br>b) YOUR DAY<br>c) YOUR WORRIES<br>d) HIS WORRIES	Yes<br><br>1<br>1<br>1<br>1	No<br><br>2<br>2<br>2<br>2	DN<br><br>3<br>3<br>3<br>3	
H88	In general, do (did) you and your (current or most recent) husband/partner discuss together how you should have sex, when, how often?	RARELY ....1<br>SOMETIMES...2<br>OFTEN............................ ....3<br>DON'T DISCUSS..4<br>REFUSED/NO ANSWER.......................................5				
H89	In your relationship with your (current or most recent) husband/partner, how often would you say that you quarrelled?<br>Would you say rarely, sometimes or often?	RARELY ....1<br>SOMETIMES...2<br>OFTEN...3<br>DON'T KNOW/DON'T REMEMBER ....4<br>REFUSED/NO ANSWER ....5<br>Didn't quarrel at all ---------------------------------------6				
L90	Did you test for HIV	Yes....................................1<br>No......................................2<br>REFUSED/NO ANSWER…….3				
	**ASK HIV positive Only. GO to question L98 if women is HIV negative**					
H91	Did you tell your husband or partner about your HIV	Yes...1				If 1

	test result?	No...2<br>REFUSED/NO ANSWER…………3	Go L93
L92	Are you planning to tell your husband/partner about your HIV test result?	YES...1<br>NO..2<br>REFUSED/NO ANSWER…………………………3	
L93	How long did it take you to disclose your HIV test results to your husband/partner?	Within three days...1<br>Within a week ....2<br>Within a month ....3<br>Within three months ....4<br>Within six months ....5<br>Over six months..6	
L94	What was his reaction after telling him or after he knew your HIV status?	Helped me..1<br>Shouted at me..2<br>Supported me...3<br>Violence............... ....4<br>Emotional violence...5<br>Thought about his HIV status ....6<br>Asked about my sexual history........................7<br>consulted the doctor/nurse.............................8<br>Threatened to beat me.....................................9<br>Threatened rejecting me.................................10<br>Rejected me...11<br>Withdrew sexual intercourse.............................12<br>Took other sexual partners...............................13<br>I don't know..14<br>Was happy...15<br>Others..16	
L95	Do you think your relationship with your partner changed for the better or for the worse or did not change?<br>after telling him your HIV status?	Better..1<br>Worse...2<br>Nothing changed..3	
L96	Did you tell any other person about your HIV status?	YES...1<br>NO..2<br>Refused/No answer..9	
L97	What type of support did you receive from him/her?	Counselling..1<br>Money...2<br>Information...3<br>Medicines/drugs...4<br>Others...5<br>Nothing...6	
	**SECTION 9 COMPLETION OF INTERVIEW**		
L98	We have now finished the interview. Do you have any comments, or is there anything else you would like?<br>--<br>--<br>--		

L99	I have asked you about many difficult things. How has talking about these things made you feel.....?<br>WRITE DOWN ANY SPECIFIC RESPONSE GIVEN BY RESPONDENT<br>____<br>**Finish**	GOOD/BETTER.................1<br>BAD/WORSE ....2<br>SAME/ NO DIFFERENCE ….. 3

Thank you very much for your participation!
