# Supplementary material for: Intimate partner violence against women living with and without HIV, and the associated factors in Wolaita Zone, Southern Ethiopia: A comparative cross-sectional study
Source: PLoS One. 2019 Aug 23;14(8):e0220919. doi: 10.1371/journal.pone.0220919 (PMC6707594; doi:10.1371/journal.pone.0220919)
Supplement: S2 File — (PDF) [file pone.0220919.s002.pdf]

<u>ስለእናቶች ጤና መጠይቅ፡</u> ጤና ይስጥልኝ? ስሜ ------------------------ ይባላል። እኛ በወላይታ ዞን ላይ ስለ እናቶች ጤና እና የሕይወት ተሞክሯቸዉን ለመማር የዳሰሳ ጥናት እያካሄድን እንገኛለን። እርስዎ በዚህ ጥናት ለመሳተፍ እንደ ሎቶሪ ዕጣ በዕድል ተመርጠዋል።እርስዎ የሚሰጡኝ መረጃ በሙሉ በሚስጥር እንደሚያዝ ለማረጋገጥ እፈላጋለሁ። የእርስዎ ስም በምንም ተዓምር በተመራማሪ አይያዝም /አይመዘገቡም። በማንኛዉም ሰዓት መጠይቁን /ዉይይቱን ለማቋረጥ ሙሉ መብት አለዎት። በተጨማሪም ማንኛዉንም ለመመለስ የማፈልጉትን መጠይቅ የመዝለል መብት አለዎት። አንዳንድ መጠይቆች ትንሽ ልከብዱ ይችላሉ። **የምርምሩ ዋና ዓላማ እና ጥቅም** ፡ እናቶች ላይ ከትዳር አጋር /ጓደኛ የሚደርስ ትንኮሳ/ግጭትና ከግጭቱ ጋር ተያያዥ ያላቸዉን ነገሮች ለመፈተሸ እና የአከባቢዉን መረጃ ለማደራጀት ከትዳር አጋር /ጓደኛ የሚደርስ ትንኮሳ/ግጭት ለመከላከል የሚሠራዉን ፕሮግራሞች ይረዳ ዘንድ ነዉ። በምርምሩ መሰተፍ ለተሳታፊዎች ቀጥተኛ የሆነ ጥቅም አያስገኝም። ቢሆንም ለተሳታፊዎች የቁስላቸዉን ታርክ ለመወያየት ምቹ ዕድል ይፈጥራል። **የምርምሩ ጉዳት :** በዚህ ምርምር ፕሮጀክት መሰተፍ የሚያሰምምዎትን የትንኮሳ ልምድ ማካፈሉ ምቹ ላይሆን ይችላል። በጣም ትንሽ የሚንገምተዉ ጉዳት ሊኖረዉ ይችላል።

አድራሻ እና ጥያቄ : ጥያቄ ካሎት ልጠይቁኝ ይችላሉ። ተጨማሪ መረጃ ካስፈለጎት ወይንም ከጥናቱ ለመዉጣት ከፈለጉ የምርምሩን ባለበት አቶ መንግሥቱ መስቀሌ በስልክ ቁጥር (+251913177996 ወይም በኢ-ሜል አድራሻ፡ <u>mengistu77@gmail.com</u>) ደዉለዉ ማናገር ይችላሉ። Biomedical Research Ethics Administration: Email: BREC@ukzn.ac.za Tel: 27 31 2604769 - Fax: 27 31 2604609.

<u>የስምምነት ቅጽ፡</u> በዚህ ጥናት የመሳተፍ ዉሳኔ የእርስዎ ነዉ።እታች ያለዉ ፊርማዎት ይህንን የስምምነት ቅጽ ማንበቦን ወይንም ሌላ ሰዉ እንዳነበበሎት እና በዚህ ጥናት ለመሳተፍ መወሰንዎን ያሳያል።ወደፊት ጥናቱን ለማቋረጥ ከፈለጉ በቀላሉ ያሳዉቁኝና በየትኛዉም ጊዜ ማቋረጥ ይችላሉ። ይህንን መጠይቅ ለማጠናቀቅ 25-30 ደቂቃ ልፈጅ ይችላል።ጥያቄዉን ለመጠየቅ ይስማማሉ? ከተሰማሙ ብቻ ይጠይቁ፡

የተሳታፊዉ ፊርማ ቀን

-------------------------------- -------------------------

የምስክር ፊርማ

1.-------------------------------------- ....

2. --- --

የመረጃ ሰብሳቢ ስምና ፊርማ

-- ---------------------------------

የሱፊርቫይዘር ስም ና ፊርማ

--- --

የተጀመረበት ሰዓት፡-------------------------------- ያለቀበት ሰዓት፡ --------------------------

ክፍል 1. ማህበራዊ እና ኢኮኖሚያዊ ነገሮች

No	ጥያቄ	መልስ	ዝለል
	የደንበኛዉ ዓይነት<br>**(በመረጃ ሰብሳቢ የሚለይ)**	1 .″ART′′ ደንበኛ<br>2. ኤች አይ ቭ ተመርምሮ ዉጤቱ ነፃ የሆነ	
S1	ዕድሜ ስንት ነዉ አሁን?	ዕድሜ በሙሉ ዓመት----------------	
S2	መኖሪያ ቦታ የት ነዉ?	1.ከተማ 2. ገጠር	
S3	የአሁኑ ጋብቻ ሁኔታ እንዴት ነዉ?	1. አሁን ያገባ/ ከወንድ ጋር የሚኖር → ወደ 7<br>2. ከወንድ ጋር የሚኖር ግን ያላገበች → ወደ 7<br>3. ባሁኑ ጊዜ መደበኛ ጓደኛ ያላት ግን በተለያየ ቦታ → ወደ 7 ይህዱ<br>4. ባሁኑ ጊዜ ያላገበች ወይም ከወንድ ጋር የማትኖር	ወደ 7<br>ወደ 7<br>ወደ 7 ይህዱ
S4	ከዚህ በፊት አግብተዉ ወይም ወንድ ጋር ኖረዉ ያዉቃሉ?	1. አዎን፣ አግብቻያለሁ<br>2. አዎን ከወንድ ጋር ኖሪያለሁ ግን አላገባሁም<br>3. አይደለም	
S5	የመጨረሻ ትዳራችሁ ከባለቤትዎ ጋር ተፋቱ፤ ተለያዩ፤ ወይንም ባለቤትዎ ሞተዋል?	1. ተፋተዋል 2. ተለያይተዋል<br>3. ባለበቴ ሞተዋል → ወደ 7 ይህዱ	3 ከሆነ<br>ወደ 7 ይህዱ
S6	የትዳርዎ መፋታት/መለያየት የተቀሰቀሰዉ/የተጀመረዉ በማን ነዉ?	1. ተሳታፊ 2. ባለቤት /ጓደኛ 3. ሁለቱም<br>4.ሌላ	
S7	ስንት ጊዜ አግብተዋል /ከወንድ ጋር ኖሩዋል?	የጋብቻ ቁጥር ---------------------<br>ፈቃደኛ አለመሆን፤መልስ የለም.......8	
S8	ከእርስዎ ጋር ግንኙነት ስያደርግ ባለቤትዎ ሌላ ምስት አለዉ?	1.አዎን 2. አይደለም 3. አላዉቅም ;አላስታዉስም<br>4. ፈቃደኛ አለመሆን	**2,3,4 ከሆነወደ 11ይህዱ**
S9	ከእርስዎ ጭምር ባለቤትዎ ስንት ምስቶች አሉት?	የምስቶች ብዛት፡--------------- ፈቃደኛ አለመሆን......8	

S10	እርስዎ አሁን/ድሮ ስንተኛ ምስት ነበሩ?<br>የመጀመሪያ፤ሁለተኛ ወይም......ስንት	ደረጃ በቁጥር፤------ፈቃደኛ አይደሉም ፤ መልስ የለም......8	
S11	ያሁኑን ባለቤቶን ለማግባት ማን መረጠ ?	1. ሁለታችን መረጥን<br>2. ተሳታፊ/እኔ<br>3. የእኔ ቤተሰብ መረጠ<br>4. ቤተክርስቲያን<br>5. ባለቤቴ መረጠ<br>6. አላዉቅም 7. ፈቃደኛ አይደለም	
S12	ያሁኑን ጋብቻ ከመፈጸማችሁ በፊት ካሁኑ ወይም ከቅርብ ጊዜ የጋብቻ አጋርዎን ለማግባት እንደሚፈልጉ ጠይቀዋል?	1. አዎን 2. አይደለም<br>3. አላዉቅም/አላስታዉስም<br>4. ፈቃደኛ አይደለም/መልስ የለም	
S13	የእርስዎ ጋብቻ የጥሎሽ ክፊያ ያካተቴ ነበር?	1. አዎን/ ለባል የተሰጠ 2. አዎን /ለሴት የተሰጠ<br>3. አይደለም 4. አላዉቅም	**3, 4 ከሆነ ወደ 15 ይህዱ**
S14	ሁሉም ጥሎሽ ተከፍሏል ወይም ገና ልከፈል የቀረ አለ?	1. ሁሉም ተከፍሏል 2. በከፍል ተከፍሏል<br>3 .ምንም አልተከፈለም 4. አላዉቅም	
S15	የቤተሰበ ብዛት ስንት ነዉ?	-------------------------------------	
S16	ሐይማኖትዎ ምንድን ነዉ?	1.ኦርቶዶክስ 2.ሙስልም 3.ፐሮተስታንት 4.ካቶልክ<br>5.ባህላዊ 6 . ሐዋሪያት 7. ሌላ ካለ ይገለጽ	
S17	ብሔሮ ምንድን ነዉ?	1.ወላይታ 2.አማራ 3. ዳዉሮ 4.ጉራጌ<br>5.ኦሮሞ 6. .ሌላ ካለ ይገለጽ --	
S18	ሥራዎ ምንድን ነዉ? (ለሴት)	1. የቤት እሜበት 2. ነጋዴ 3.ተማሪ<br>4. የመንግሥት / መግሥታዊ ባልሆነ ድርጅት ሠራተኛ<br>5. ቀን ሠራተኛ 6. ሌላ ካለ ይገለጽ --	
S19	የባለቤትዎ ሥራ ምንድን ነዉ?	1. ገበሬ 2. የመንግሥት ሠራተኛ 3. ነጋዴ 4. ቀን ሠራተኛ 5. መግሥታዊ ባልሆነ ድርጅት ሠራተኛ 6. ሌላ	
S20	መደበኛ ትምህርት ተከታትለዋል?	1.አዎን 2. አይደለም → **2 ከሆነ ወደ 22 ይህዱ**	

S21	ያጠናቀቁት ከፍተኛ ክፍል ስንት ነዉ?	ያጠናቀቁት ክፍል......................................	
S22	ባለቤትዎ መደበኛ ትምህርት ተከታትለዋል?	1.አዎን 2. አይደለም → **2 ከሆነ ወደ 23 ይህዱ**	
S23	ባለቤትዎ ያጠናቀቀዉ ከፍተኛ ክፍል ስንት ነዉ?	ያጠናቀቀዉ መደበኛ ክፍል.........................	
S24	ወራዊ ገቢ ምን ያህል ነዉ?	በኢትዮጵያ ብር: --------------------------------------	
S25	**በቤትዎ ዉስጥ የሚሠራ ....አለ** ....: [አዎን ከሆነ '**1**' አይደለም ከሆነ '**0**' ጻፍ.]<br><br>W1. ተሌቭዥን: |___| W2. ሬድዮ: |___| W3. ሳተላይት ዲሽ: |___| W4. ሞባይል ስልክ: |___| <br>W5. የቤት ስልክ [ ] W6. ጠረጴዛ [ ] W7. ወንበር [ ] W8. አልጋ /የጥጥ/እስፎንጅ/ስፍርንግ [ ]<br><br>W9. ኤሌክትሪክ ምጣድ:|___| W10. ብስክሌት:|___| W11. ሞቶር ሳይክል/ባጃጅ :|___| W12. ፍሪጅ: |___| <br>W13. ኤሌክትሪስቲ/መብራት [ ] W14. ትራክተር/መኪና |___| 15 .የእርሻ መሬት/ የከተማ መሬት [ ]<br><br>W16. የእንጀራ ምጣድ(የማገዶ) [ ] W. 17 ኤሌክትርክ ስቶቭ/ ምድጃ [ ]		

ክፈል 2፡ ስያስቡ ያሁኑ ወይም የቅርብ ጊዜ ባለቤትዎ/ ጓደኛዎት የሚከተለዉ ሃሳብ ባጠቃላይ እዉነት ነዉ ብለዉ ያሰባሉ ? **(ዉሳነ የመወሰን ያለዉ መብት)**

S.No	መጠይቅ	አዎን	አይደለም	አላዉቅም
D27	ጓደኛዎትን እንዳያዩ /እንዳይጎበኙ ለማድረግ ይሞክራል?	1	2	8
D28	ወላጅ ቤተሰብ ጋር እንዳትገናኝ ለመቆጣጠር ይሞክራል?	1	2	8
D29	ሁል ጊዜ የምትዉልበትን/የምትገኝበትን ለማወቅ ይፈልጋል?	1	2	8
D30	እርስዎን ለሰዉ ከሚያደርገዉ **በልዩነት ይጎዳል** ወይም እርስዎን ይረሳል ?	1	2	8
D31	ከሌላ ወንድ ጋር ካወሩ ይቆጣል?	1	2	8
D32	ታማኝ አይደለሽም ብሎ ሁል ጊዜ ይጠራጠራል?	1	2	8

D33	የህክምና አገልግሎት ለማግኘት የእሱን ፈቃድ እንዲታገኚ ይፈልጋል?	1	2	8

S.N.	ጥያቄ<br>**<u>ስሜታዊ ጥቃት/ስነልቦናዊ</u>**	A) አዎን ከሆነ"B"ይቀ ጥሉ።አይደለ ምከሆነወደ ሚቀጥለዉ ይሂዱ	B) ይህ ባለፉት 12 ወራትዉስጥ ተከስቷል?(አዎን ከሆነ "C"ብቻ ይጠይቁ፡አይደለ ም ከሆነ "D" ን ብቻ ይጠይቁ)	C)ባለፉት 12 ወራት ዉስጥ ይህ የተከሰተዉ አንዴ ፤ጥቂት፤ ወይም ብዙ ጊዜ ነዉ? (C ን ከመለሱ ወደ ሚቀጥለዉ ይሂዱ)	D)ከ12 ወራት በፊት ይህ የተከሰተዉ አንዴ ፤ጥቂት፤ ወይም ብዙ ጊዜ ነዉ?

የሚከተለዉ ጥያቄ ለብዙ እናቶች የሚከሰትና የእርስዎ ያሁኑ የትዳር ጓደኛ ወይም ሌላ ጓደኛ በእርስዎ ላይ ስላደረሰዉ ነገር ነዉ። የእርስዎ ያሁኑ የትዳር ጓደኛ ወይም ሌላ ጓደኛ በየትኛዉም ጊዜ፤

		አዎን	አይደለም	አዎን	አይደለም	አንዴ	ጥቂት	ብዙ ጊዜ	አንዴ	ጥቂት	ብዙ ጊዜ
E34	እርስዎን ተሳድቧል ወይም ስለእራስዎ መጥፎ እንዲሰማዎት አድርጓል?	1	2	1	2	1	2	3	1	2	3
E35	በሰዉ ፊት አንቋሽሿል/ዝቅ አድርጓል?	1	2	1	2	1	2	3	1	2	3
E36	ሆን ብሎ በነገሮች ማስፈራራት (ለምሳሌ ወደ አንቺ በማየት፤ ዕቃዎችን በማጮህ?	1	2	1	2	1	2	3	1	2	3
E37	እርስዎን ወይም እርስዎ የሚሳሱለትን ሰዉ ለመጉዳት ማስፈራራት?	1	2	1	2	1	2	3	1	2	3

S.N.	ጥያቄ<br>እርሱ ወይም ሌላ ጓደኛ.... **<u>አካላዊ ጥቃት</u>** ከሚከተለዉ አድርሷል?	A) አዎን ከሆነ "B"ይቀጥሉ። አይደለም ከሆነ ወደሚቀጥለዉ ይሂዱ		B) ይህ ባለፉት 12 ወራት ዉስጥ ተከስቷል? (አዎን ከሆነ "C''ብቻ ይጠይቁ. አይደለም ከሆነ "D" ን ብቻ ይጠይቁ)		C)ባለፉት 12 ወራት ዉስጥ ይህ የተከሰተዉ አንዴ ፤ጥቂት፤ ወይም ብዙ ጊዜ ነዉ? (Cን ከመለሱ ወደ ሚቀጥለዉ ይሂዱ)			D)ከ12 ወራት በፊት ይህ የተከሰተዉ አንዴ ፤ጥቂት፤ ወይም ብዙ ጊዜ ነዉ?		
		አዎን	አይደለም	አዎን	አይደለም	አንዴ	ጥቂት	ብዙ ጊዜ	አንዴ	ጥቂት	ብዙ ጊዜ
P38	መትቶሻል ወይም እርስዎ ላይ የወረወሩት ነገር ጉዳት አድርሷል?	1	2	1	2	1	2	3	1	2	3
P39	ገፍቷል ወይም ፀጉር ጎትቷል?	1	2	1	2	1	2	3	1	2	3
P40	በቡጢ /በቦክስ መቷል ወይም በሌላ ነገር ጎድቷል?	1	2	1	2	1	2	3	1	2	3
P41	በእርግጫ መቷል፤ጎትቷል ወይም መቷል?	1	2	1	2	1	2	3	1	2	3
P42	በሚያቃጥል ነገር ሰዉነቶን ሆን ብሎ አቃጥሏል?	1	2	1	2	1	2	3	1	2	3
P43	መሣሪያ(ጠመንጃ፤ሹጉጥ ወዘተ እጠቀማለሁ ብሎ አስፈራርቷል፤ወይም መሣሪያዉን፤ቢላዋን፤ወይንም ሌላ መሣሪያን እርስዎን ለመጉዳት ተጠቅሟል?	1	2	1	2	1	2	3	1	2	3

ተ.ቁ	ጥያቄ<br>እርሱ ወይም ሌላ ጓደኛ....<br>**<u>ስለ ወስባዊ ጥቃት</u>** ከሚከተለዉ?	A) አዎን ከሆነ "B"ይቀጥሉ፡፡ አይደለም ከሆነ ወደሚቀጥለ ዉ ይሂዱ	B) ይህ ባለፉት 12 ወራትዉስጥ ተከስቷል? (አዎን ከሆነ"C''ብቻ ይጠይቁ. አይደለም ከሆነ "D" ን ብቻ ይጠይቁ)	C)ባለፉት 12 ወራት ዉስጥ ይህ የተከሰተዉ አንዴ ፤ጥቂት፤ ወይም ብዙ ጊዜ ነዉ? (Cን ከመለሱ ወደ ሚቀጥለዉ ይሂዱ)	D)ከ12 ወራት በፊት ይህ የተከሰተዉ አንዴ ፤ጥቂት፤ ወይም ብዙ ጊዜ ነዉ?

		አዎን	አይ ደለም	አዎን	አይደ ለም	አንዴ	ጥቂ ት	ብዙ ጊዜ	አ ን ዴ	ጥ ቂ ት	ብዙ ጊዜ
S44	እርስዎ ሳይፈልጉ ያሁኑ ትዳር አጋርዎ ፤ጓደኛዎ፤ወይም ማንኛዉም ሌላ አጋርዎ የግብረ-ሥጋ ግንኙነት እንዲያደርጉ **በአካላዊ(በኃይል) አስገድዷል**?	1	2	1	2	1	2	3	1	2	3
S45	አጋርዎ ወይም ሌላ ጓደኛ ስለሚያደርጉት ነገር እየፈሩ ነገር ግን **ሳይፈልጉ** **የግብረ-ሥጋ ግንኙነት ፈጽሟል?**	1	2	1	2	1	2	3	1	2	3
S46	አጋርዎ ወይም ሌላ ጓደኛ በኃይል አስገድዶ ፆታዊ ግንኙነት መፈጸሙ **ስብዕናዎን/ሞራሎን የነካበት** ሁኔታ አለ?	1	2	1	2	1	2	3	1	2	3

<table>
<tr><td>ተ.ቁ</td><td>መጠይቅ</td><td colspan="3"></td></tr>
<tr><td>P47</td><td>በአካላዊ ትንከሳ የተጠየቁ ማንኛዉንም ጥያቄዎች አዎን መሆናቸዉን አረጋግጥ? የጥያቄ ቁጥር P38-P43 ተመልከት.</td><td colspan="3">1. አዎን፤አካላዊ ትንከሳ አለ
2. አካላዊ ትንከሳ የለም</td></tr>
<tr><td>S48</td><td>ፆታዊ ትንኮሳ የተጠየቁ ማንኛዉንም ጥያቄዎች አዎን መሆናቸዉን አረጋግጥ? የጥያቄ ቁጥር S44-S46 ተመልከት.</td><td colspan="3">1. አዎን ፆታዊ ትንኮሳ አለ
2. ፆታዊ ትንኮሳ የለም</td></tr>
<tr><td>I49</td><td>ነፍሰ ጡር ሆነዉ ያዉቃሉ?</td><td colspan="3">1. አዎን 2. አይደለም 2 ከሆነ ወደ 52 ይህዱ</td></tr>
<tr><td>I50</td><td>ነፍሰ ጡር እንደነበሩ ነግረዉኛል ። ምናልባት በዛን ጊዜ በአጋሮ ወይም በማንኛዉም ጓደኛ የተመቱበት/የተደበደቡበት ጊዜ ነበረዎት ?</td><td colspan="3">1. አዎን 2. አይደለም → 2,3,4 ከሆነ ወደ 52 ይህዱ
3. አላዉቂም/ አላስታዉስም
4.ለመመለስ ፈቃደኛ አልሆኑም/መልስ የለም</td></tr>
<tr><td>I51</td><td>በደረሰዉ አካላዊ ትንከሳ የተነሳ ዉርጃ አጋጥሟል ?</td><td colspan="3">1.አዎን 2. አይደለም</td></tr>
<tr><td rowspan="11">52</td><td rowspan="11">አካላዊ ጉዳት፤ስንጥቅ፤ጭረት ፤ወለምታ፤ቃጠሎ ወይም የአጥንት ስብራት ከትዳር አጋርዎ ፤ጓደኛዎ፤ወይም ማንኛዉም ሌላ አጋርዎ ከዚህ ከፍ ብሎ በተነጋገርናቸዉ ጉዳዮች ምክንያት ደርሶበት ያዉቃል?

1. አዎን
2. አይደለም

አይደለም ከሆነ ወደ 53 ይህዱ</td><td></td><td>አዎን</td><td>አይደለም</td></tr>
<tr><td>1. የተቆረጠበት፤የተበሳ ቁስል፡ ንክሻ</td><td>1</td><td>2</td></tr>
<tr><td>2. የተቧቸጨረ፤መገሸለጥ፤ መላላጥ</td><td>1</td><td>2</td></tr>
<tr><td>3. ወለምታ፤ ዉልቃት</td><td>1</td><td>2</td></tr>
<tr><td>4. ቃጠሎ</td><td>1</td><td>2</td></tr>
<tr><td>5. የተበሳ ቁስል፤ በጥልቅ</td><td>1</td><td>2</td></tr>
<tr><td>6. የተቆረጠበት፤መድማት</td><td></td><td></td></tr>
<tr><td>7. የተጎዳ የጆሮ ፋምቡር፤የዓይን ቁስል</td><td>1</td><td>2</td></tr>
<tr><td>8. ስብራት፤የአጥንት ስብራት</td><td>1</td><td>2</td></tr>
<tr><td>9. የጥርስ ስብራት</td><td>1</td><td>2</td></tr>
<tr><td>10. ሌላ</td><td></td><td></td></tr>
<tr><td>I53</td><td>በሕይወትዎ ከትዳር አጋርዎ ፤ጓደኛዎ፤ወይም ማንኛዉም ሌላ አጋርዎ ምን ያህል ጊዜ ቆስለዋል/ተጎድተዋል?
ምን ያህል ጊዜ?</td><td colspan="3">1. ምንም → 1, እና 5 ከሆነ ወደ 59 ይህዱ
2. አንዴ/ሁለቴ
3. ብዙ ጊዜ (3-5) ጊዜ
4. ብዙ (ከ5 በላይ) ጊዜ
5. አላዉቂም/ አላስታዉስም → 5 ከሆነ ወደ 59 ይህዱ
6. ለመመለስ ፈቃደኛ አልሆኑም/መልስ የለም</td></tr>
<tr><td>I54</td><td>ይህ ባለፋት 12 ወራት ዉስጥ ተከስቷል ?</td><td colspan="3">1. አዎን 2. አይደለም 3. አላዉቅም/ አላስታዉስም
4. ለመመለስ ፈቃደኛ አልሆኑም/መልስ የለም</td></tr>
<tr><td>I55</td><td>ስለዚህ ጉዳት የጤና አገልግሎት አግኝቷል ?</td><td colspan="3">1. አዎን 2. አይደለም</td></tr>
</table>

I56	ስለዚህ ጉዳት እርዳታ ለማግኘት ፈልጎ ነበር?	1. አዎን<br>2. አልፈለጉም
I57	ይህንን የባለቤትዎን አካላዊ የትንኮሳ ባህሪን ለማን ነገሩ ?	1. ለማንም አልነገርኩም 2. ለጓደኛ 3. ለቤተሰብ<br>3. ለወንድም ወይም ለእህት 5. አጎት አክስት<br>6. ለባል/ ለወላጅ ቤቴሰብ 7. ለልጆች 8. ለጎረበት 9. ለፖሊስ<br>10. ለዶ/ር / ጤና ባለሙያ 11. ለሃይማኖት መሪዎች 12. ለአማካሪዎች<br>13. ለ NGO /ለሴቶች ማህበር 14. ለመንደር መሪዎች<br>15. ለህግ ማህበር 16. ለሌላ
I58	ለመርዳት የሞከረ ሰዉ ነበር?	1. የለም 2. ለጓደኛ 3. ለቤተሰብ<br>4. ለወንድም ወይም ለእህት 5. አጎት አክስት<br>6. ለባል/ ለወላጅ ቤቴሰብ 7. ለልጆች 8. ለጎረበት 9. ለፖሊስ<br>10. ለዶ/ር / ጤና ባለሙያ 11. የሃይማኖት መሪዎች 12. ለአማካሪዎች<br>13.ለ NGO ለሴቶች ማህበር 14. ለመንደር መሪዎች<br>13. ለህግ ማህበር 16. ለሌላ

አካላዊ ትንኮሳ የደረሳቸዉ ሴቶች

W59	ድብዴባ በደረሰብዎት ጊዜ **<u>መልሰዉ በእሱ</u>** ላይ አካላዊ ድብዴባ አድርጓል ወይም እራስዎን ተከላክለዋል?<br>**1.አዎን**<br>**2.አይደለም**<br>አዎን ከሆነ : ምን ያህል ጊዜ?	1. አንዴ ወይም ሁለቴ<br>2. ብዙ ጊዜ / አብዘኛዉ ጊዜ<br>3. አላዉቅም / አላስታዉስም<br>4. አንቢ / መልስ የለኝም
W60	ባለቤትዎ እርስዎን ሳይመታ እርስዎ የእሱን አካል መትተዉ ወይም ተችተዉ ያዉቃሉ?<br>1.**አዎን**<br>**2.አይደለም**<br>አዎን ከሆነ ምን ያህል ጊዜ ?	1. አንዴ ወይም ሁለቴ<br>2. ብዙ ጊዜ / አብዘኛዉ ጊዜ<br>3. አላዉቅም / / አላስታዉስም<br>4. አንቢ / መልስ የለኝም

ክፍል: የሕይወት ዘመን የትንኮሳ ልምድ ከዘመድ፤ከሌላ ከሚያዉቁት ሕዝብ፤ እና/ወይም ከእንግዳ

S.No.	ጥያቄ		የተከበቡትን ብቻ ጠይቅ ምን ያክል ጊዜ ነዉ ይህ የተከሰተዉ?		
			አንዴ ወይም ሁለቴ	ጥቅት ጊዜ	አብዘኛዉ ጊዜ
W61	**<u>ከ15 ዓመት በኋላ</u>** <u>ያሁኑ ወይም የድሮ ጓደኛ</u>፤ ወይም **ከባለበትሽ ዉጭ አካልሽን የደበደቤ** ወይም በሌላ ነገር የመታሽ ሰዉ አለ?<br>**1.አዎን**<br>**2. አይደለም ,** አዎን ከሆነ ማን አደረገ?<br>ፈትሽ: ዘመድስ እንዴት ነዉ?	1.ማንም	1	2	3
		2. አባት	1	2	3
		3. የእንጀራ አባት	1	2	3
		4.ሌላ ወንድ የቤት አባል	1	2	3
		5. ሴት የቤት አባል	1	2	3
		6. አስተማሪ	1	2	3

	ትምህርት ቤት ወይም ሥራ ላይ ያለዉ ሰዉስ?<br>ጓደኛ ወይም ጎረቤት?<br>እንግዳ ወይም ሌላ ማንኛዉም ሰዉ?	7.ፖሊስ/ ወታደር	1	2	3
		8. የቤቴሰብ ወንድ ጓደኛ	1	2	3
		9. የቤተሰብ ሴት ጓደኛ	1	2	3
		10. የወንድ ጓደኛ	1	2	3
		11. እንግዳ	1	2	3
		12. በሥራ ላይ ያለዉ ሰዉ	1	2	3
		13. ቄስ/የሐይማኖት	1	2	3
		14. መሪ	1	2	3

ክፍል: ከዘመድ፤ከሚያዉቁት ሌላ ሰዉ፤ ወይም ከእንግዳ በሕይወት ዘመን የሚደርስ ትንኮሳ

S. No.	ጥያቄ	ማን ነዉ ያደረገዉ?	የተከበቡትን ብቻ ጠይቅ ምን ያክል ጊዜ ነዉ ይህ የተከሰተዉ?		
			አንዴ / ሁለቴ	ጥቅት ጊዜ	አብዘኛዉ ጊዜ
S62	<u>**ከ15 ዓመት በፊት**</u>, ከቤተሰብ አባላት ማናቸዉም ለግብረ-ሥጋ ግንኙነት እርስዎን ነክቷል ወይም እርስዎ <u>**ባይፈልጉም የግብረ-ሥጋ ግንኙነት እንዲያደርጉ አድርጓል?**</u><br><br>1.**አዎን**<br>**2.አይደለም**<br>አዎን ከሆነ: ማን ነዉ ያደረገዉ? አዎን ከሆነ ወይም አይደለም ከሆነም ይቀጥሉ:<br>በትምህርት ቤት አንዴት ነዉ?<br>ጓደኛ ወይም ጎረበት እንዴት ነዉ?<br>ማንኛዉም ሰዉ በእርስዎ ላይ ይህንን አድርጓል?<br>አዎን ከሆነ: ማነዉ ያደረገዉ?	1.ማንም	1	2	3
		2. አባት	1	2	3
		3. የእንጀራ አባት	1	2	3
		4. ሌላ ወንድ የቤተሰብ አባል	1	2	3
		5. ሴት የቤተሰብ አባል	1	2	3
		6. አስተማሪ	1	2	3
		7.ፖሊስ/ ወታደር	1	2	3
		8. ወንድ የቤተሰብ ጓደኛ	1	2	3
		9. ሴት የቤተሰብ ጓደኛ	1	2	3
		10. ወንድ ጓደኛ	1	2	3
		11.እንግዳ	1	2	3
		12. በሥራ ላይ ያለዉ ሰዉ	1	2	3
		13. ቄስ/ የሐይማኖት	1	2	3
		14. መሪ	1	2	3

S.N.	**ጥያቄ**	መልስ	ዝለል
L63	አካላዊ ወይም ፆታዊ ጥቃት ባጋጠሞት ጊዜ የሁለታችሁ ግንኙነት ምን ሆነ?	1. በጊዜያዊነት ግንኙነቱንአቋሚያለሁ<br>2. በቋሚነት ግንኙነቱን አቋሚያለሁ<br>3. ጥቃት አላጋጠመኝም 4.ሌላ	**3 Go H65**
L64	አካላዊ ወይም ፆታዊ ጥቃት ያስከተለዉ ዉጤት ምንድን ነዉ?	1. ምንም<br>2. እራስህን መጉዳት/ ማዋረድ<br>3. የጤናና ህክምና ችግር<br>4. ከህብረተሰብ መገለል<br>5. ለመሠረታዊ ፍላጎቶች መድረሱ መቀነስ	
H65	አሁን ወይም በፊት ባለቤትዎ አልኮል ይጠጣል?	1. አዎን 2. አይደለም	2 ከሆነ ወደ 69 **ይህዱ**
H66	ምን ያህል ጊዜ ነዉ አልኮል የሚጠጣዉ?	1. ሁል ቀን ወይም ለሁል ቀን ተቀራራቢ<br>2. በሳምንት አንዴ / ሁለቴ<br>3. በወር 1-3 ጊዜ<br>4. አልፎ አልፎ<br>5 አላዉቅም/አላስታዉስም<br>6. ፈቃደኛ አይደለሁም/መልስ የለኝም	

H68	ባለፉት 12 ወራት ዉስጥ የባለቤትዎ አልኮል መጠጣት ጋር በተያያዘ ያጋጠመዎት ችግር ከሚከተለዉ የትኛዉ ነዉ?		አዎን	አይደለም	
		A) የገንዘብ ችግር	1	2	
		B) የጤና ችግር	1	2	
		C)ከቤተሰብ እና ጓደኛ ጋር ፀብ	1	2	
		D)ከኃላፊዎች ጋር ችግር (የሆተል ባለቤት/ፖሊስ ወዘተ,)	1	2	
		E) ችግር የለም F. ሌላ ይገለጽ	1	2	
L69	ባለፋት አራት ወራት ዉስጥ ራስዎን ለማጥፋት **አስበዎት** ያዉቃሉ?	1 .አዎን 2.አይደለም 3 . አላዉቅም<br>4 . ፈቃደኛ አይደለሁም/ መልስ የለኝም			
L70	ራስዎን ለማጥፋት **ሞክረዉ** ያዉቃሉ?	1 .አዎን 2.አይደለም 3 . አላዉቅም<br>4 . ፈቃደኛ አይደለሁም/ መልስ የለኝም			
H71	ያሁኑን ወይም የቅርብ ጊዜ ጓደኛዎትን/ባለቤቶን **ኮንዶም እንዲጠቀም** ጠይቀዉ ያዉቃሉ?	1 .አዎን 2.አይደለም 3 . አላዉቅም<br>4 . ፈቃደኛ አይደለሁም/ መልስ የለኝም			
H72	ያሁኑ ወይም የቅርብ ጊዜ ጓደኛዎ/ባለቤትዎ ኮንዶም **ለመጠቀም እንቢ ብሎ** ያዉቃል?	1 .አዎን 2.አይደለም 3 . አላዉቅም 4 .ፈቃደኛ አይደለሁም/ መልስ የለኝም **2-4ከሆነ ወደ 74 ይህዱ**			
H73	በምን ዓይነት መንገድ ነዉ ኮንዶምን **ለመጠቀም እንቢ ማለቱን የገለጸዉ?**	ለመጠቀም እንቢ ማለቱን ነገረኝ .... A<br>ጮኼብኝ/ ተቆጣኝ .... B<br>ለመምታት አስፈራራኝ C			

	ሁሉንም ምላሾች ያክብቡ	ከቤት ለማዉጣት አስፈራራኝ..............D<br>መታኝ/አካሌን ተነኮሰኝ........................E<br>ኮንዶሙን አጠፋ ....F<br>ታማኝ እንዳልሆንኩኝ ከሰሰኝ/ጥሩ ሴት እንዳልሆንኩኝ.. G<br>ሳቀብኝ/በጥሩ አላየኝም......................... H<br>አስፈላጊ እንዳልሆነ ነገረኝ......................I<br>ሌላ ________________________X	
L74	አሁን ስለመጀመሪያ ጊዜ ስለፈፀሙት ግብረ-ሥጋ ግንኙነት ልጠይቆት። **በምን ዕድሜ/መቼ የግብረ-ሥጋ ግንኙነት ጀመሩ?**	ዕድሜ በሙሉ ዓሜት: ---------------------------	
L75	ከሚከተለዉ የትኛዉ ነዉ እርስዎ በመጀመሪያ ጊዜ የግብረ-ሥጋ ግንኙነት ስያደርጉ የተፈጸመዉን ድርጊት የሚገልጸዉ?	1 .እኔ ፈቃደኛ ነበርኩ 2. አሳምነዉኝ ነበር<br>3 .ተታልዬ ነዉ 4. ተገድጄ ነበር<br>5.ተደፍሪያለሁ	
L76	ይኼ የሆነዉ ከማን ጋር ነበር?	1. ባል 2.ወንድ ጓደኛ 3.አስተማሪ<br>4. አባት/ቤተሰብ አባል 5. የትምህርት ቤት ወንድ/ የአከባቢ 6. የጓደኛ ቤተሰብ<br>7. ዘመድ 8.እንግዳ/የማይታወቅ ሰዉ 9. ሌላ	
L77	እርስዎ ከዛ ሰዉ ጋር የግብሬ-ሥጋ ግንኙነት ስፈጽሙ **<u>ለእሱ/ ለዛ ሰዉ</u>** ስንት ዓመት ነበር?	በዓመት --------------------------------<br>A. ከእኔ በዕድሜ ያንሳል<br>B. ተመሳሳይ ዕድሜ ነበርን<br>C. 1-2 ዓመት ይበልጠኝ ነበር<br>D. 3-5 ዓመት ይበልጠኝ ነበር<br>E. 5-10 ዓመት ይበልጠኝ ነበር<br>F. ከ10 ዓመት በላይ ይበልጠኝ ነበር<br>G. ፈቃደኛ አይደለም/መልስ የለኝም	
L78	ለእናቶች ያሉዋቸዉ የግብረ-ሥጋ ግንኙነት ጓደኛ ከአንዱ ወደ ሌላዉ እናት በጣም ይለያያል።. አንዳንዶች እናት አንድ ጓደኛ ፤ለሌላ 2 ወይም ከዛ በላይ፤ ለሌላ ደግሞ ብዙ እንዳዉም ከ 50 በላይ: **በሕይወትዎ ምን ያክል ከተለያዩ ወንዶች ጋር ነዉ ግንኙነት ያደረጉት?**ፈትሽ: ብዙ ነዉ ወይም ጥቂት; እቅጩን አይደለም ለማዎቅ የሚፈላገዉ.	ጓደኞች ብዛት ....<br>አላዉቅም/ አላስታዉስም.......................1<br>ፈቃደኛ አይደለም/መልስ የለም..............2	
L79	ህፃን በነበሩ ጊዜ እናትዎ በአባትዎ ወይም በራስዋ ወንድ ጓደኛ ተመትተዉ ነበር?	1.አዎን 2. አይደለም<br>3 .ወላጆች በአንድነት አይኖሩም 4 . አላዉቅም<br>5 . ፈቃደኛ አይደለሁም/መልስ የለንም	
L80	ህፃን በነበሩ ጊዜ ይህንን **ትንኮሳ አይተዉ ወይም ሰምተዉ** ነበር?	1 .አዎን 2.አይደለም 3 . አላዉቅም<br>4 . ፈቃደኛ አይደለሁም/ መልስ የለኝም	
H81	የባለቤትዎ እናት በራሳቸዉ ባል ተመትተዉ ያዉቃሉ?	1.አዎን 2. አይደለም<br>3 .ወላጆች በአንድነት አይኖሩም 4 . አላዉቅም<br>5 . ፈቃደኛ አይደለሁም/መልስ የለንም	

H82	የቅርብ ጊዜ ባለቤትዎ/ጓደኛ ይህንን ጥቃት **አይቶ ወይም ሰምቶ ያዉቃል?**	1 .አዎን 2.አይደለም 3 . አላዉቅም<br>4 . ፈቃደኛ አይደለሁም/ መልስ የለኝም			
H83	የእርስዎ ያሁኑ ባለቤትዎ /የቅርብ ጊዜ ወንድ ጓደኛ **<u>በቤተሰቡ/ወላጅ ድብደባ</u>** ይደርስበት ነበር ወይ?	1 .አዎን 2.አይደለም 3 . አላዉቅም<br>4 . ፈቃደኛ አይደለሁም/ መልስ የለኝም			
H84	ባለቤቶት **<u>ከሌላ ወንድ ጋር አካላዊ ድብደባ ወስጥ</u>** ተሳትፎ / ተደባድቦ ያዉቅ ነበር?	1 .አዎን 2.አይደለም 3 አላዉቅም<br>4 . ፈቃደኛ አይደለሁም/ መልስ የለኝም			
A85	**<u>ክፍል: የባለቤት መደብደብ ዝንባሌ</u>**<br>የሚከተሉትን ዓረፍተ ነገሮች አነብሎታለሁ፡፡ ከወንድ ባህሪ ባለቤታቸዉን ለመምታት ጥሩ ምክንያት ልኖረዉ የሚችለዉ ከሚከተለዉ በየትኛዉ ነዉ? <u>መስማማት አለመስማማቶን የእርስዎን ሀሳብ ይንገሩ</u> ?				
		Yes	No	DN	
	A) **የቤት ሥራዋን እሱን በሚያረካ መልኩ ሠርታ ካላጠናቀቀች** ለመምታት ጥሩ ምክንያት ነዉ፡፡	1	2	3	
	B) **እሱን ካልታዘዘች** ለመምታት ጥሩ ምክንያት ነዉ፡፡	1	2	3	
	C) **እሱ ጋር የግብረ-ሥጋ ግንኙነት እንቢ ካለች** ለመምታት ጥሩ ምክንያት ነዉ፡፡	1	2	3	
	D) **ሌላ የሴት ጓደኛ እንዳለዉ ከጠየቀች** ለመምታት ጥሩ ምክንያት ነዉ፡፡	1	2	3	
	E) **እሱ ታማኝ አይደለሽም ብሎ ከጠረጠራት** ለመምታት ጥሩ ምክንያት ነዉ፡፡	1	2	3	
	F) **ታማኝ አለመሆኑዋን ካረጋገጠ** ለመምታት ጥሩ ምክንያት ነዉ፡፡	1	2	3	
A86	ያገቡ እናቶች ለባለቤታቸዉ የግብረ-ሥጋ ግንኙነት እንቢ ለማለት ከሚከተለዉ የትኛዉን ይችላሉ?				
	a) **ባለቤቷ ካላፈለገች** የግብረ-ሥጋ ግንኙነት እንቢ ለማለት ትችላለች፡፡	1	2	3	
	b) **ባሏ መጠጥ ከጠጣ** የግብረ-ሥጋ ግንኙነት እንቢ ለማለት ትችላለች፡፡	1	2	3	
	c) **እሷ ከታመመች** የግብረ-ሥጋ ግንኙነት እንቢ ለማለት ትችላለች፡፡	1	2	3	
	d) **ባሏ ካላከበራት/ካንቋሸሻት** የግብረ-ሥጋ ግንኙነት እንቢ ለማለት ትችላለች፡፡	1	2	3	
	e) **ከጋብቻ ዉጭ ሌላ ሴት ጓደኛ መኖሩን ከጠረጠረች** የግብረ-ሥጋ ግንኙነት እንቢ ለማለት ትችላለች፡፡	1	2	3	
	f) **ባለቤቷ አባለዘር በሽታ/ኤች ኤችይቭ ካለበት** የግብረ-ሥጋ ግንኙነት እንቢ ለማለት ትችላለች፡፡	1	2	3	
	**ተሳታፊዋና ባለቤቷ**				
H87	ባጠቃላይ ያሁኑ ባል /የቅርብ ጊዜ ወንድ ጓደኛ የሚከተሉትን ርዕሶች **<u>ተወያይተዉ ያዉቃሉ፡</u>**	Yes	No	DN	
	a) ስለእሱ ቀን ዉሎ ሁኔታ	1	2	3	
	b) ስለ አንቺ ቀን ዉሎ ሁኔታ	1	2	3	
	c) ስለአንቺ ጭንቀት ወይም ስሜት	1	2	3	
	d) ስለ እሱ ጭንቀት ወይም ስሜት	1	2	3	
H88	ባጠቃላይ ያሁኑ ባል /የቅርብ ጊዜ ወንድ ጓደኛ ግብረ-ሥጋ ግንኙነት እንዴት ማድረግ እንዳለባችሁ **<u>ተወያይተዉ</u>** ያዉቃሉ፤መቼ, ምን ያህል ጊዜ?	1.አልፎ አልፎ 2. አንዳንዴ 3. ሁል ጊዜ<br>4. አንወያይም<br>5. ፈቃደኛ አይደለሁም/መልስ የለም			
H89	ያሁኑ ባል /የቅርብ ጊዜ ወንድ ጓደኛ ጋር በነበራችሁ	1.አልፎ አልፎ 2. አንዳንዴ 3. ሁልጊዜ (ብዙ ጊዜ)			

	ግንኙነት ምን ያህል ጊዜ ነዉ **<u>የተደባደባችሁት?</u>**	4.አላዉቅም /አላስታዉስም 5. ፈቃደኛ አይደለሁም<br>6. አልተደባደብንም	
L90	**ኤች አይብ ተመርምረዋል?**	1.አዎን 2. አይደለም 3. ፈቃደኛ አይደለሁም/መልስ የለም	
	ደንበኛዉ ኤች አይብ ፖዜቲብ ከሆነ ብቻ ይጠይቁ። ከኤች አይብ ነፃ ከሆነ ወደ ጥያቄ 98 ይህዱ።		
H91	ስለኤች አይብ ምርመራ ዉጤት ለባለቤትዎ ነግረዋል?	1.አዎን 2.አይደለም <u>አዎን ከሆነ ወደ 93 ይህዱ</u><br>3. ፈቃደኛ አይደለሁም/መልስ የለም	
L92	ስለኤች አይብ ምርመራ ዉጤት ለባለቤትዎ ለመንገር **<u>እያቀዱ</u>** ነዉ?	1. አዎን 2. አይደለም <u>1,2,3 ወደ 96 ይህዱ</u><br>3. ፈቃደኛ አይደለሁም/መልስ የለም	
L93	ስለኤች አይብ ምርመራ ዉጤት ለባለቤትዎ **<u>ለመንገር</u>** ምን ያክል ጊዜ ፈጀዎት?	1.ሦስት ቀናት 2. አንድ ሳምንት 3. አንድ ወር<br>4. 3 ሳምንታት 5. 6 ሳምንት 6.ከ 6 ሳምንት በላይ	
L94	ባለቤትዎ የእርስዎን ኤች አይብ ምርመራ ዉጤት ከሰማ በኋላ ያሳየዉ ምላሽ እንዴት ነበር?	1.እርዳታ ሰጠኝ 2. ጮኸብኝ 3. ደገፈኝ<br>4. ጥቃት ፈጸመብኝ 5. ስነልበናዊ ጥቃት አደረሰብኝ<br>6. ስለራሱ ኤች አይብ አስተማረኝ<br>7. ስለግብረሥጋ ሕወቴን ታርክ ጠየቀኝ<br>8. ዶ/ር/ነርስ አማከራቸዉ 9. ለመምታት አስፈራራኝ<br>10. ለመተዉ አስፈራራኝ 11.ተወኝ<br>12. ግብረ-ሥጋ ግንኙነት ተወኝ<br>13. ሌላ ግብረ-ሥጋ አጋር ወሰደ 14. አላዉቅም<br>15. ደስተኛ ነዉ 16. ሌላ..........................	
L95	ከባለቤቶ ጋር ያለዉ ግንኙነት የኤች አይብ ዉጤት ከገለጹ በኋላ ወደ ጥሩ ተቀየረ ወይስ ወደ መጥፎ?	1.ጥሩ 2.መጥፎ 3.ምንም የተቀየረ ነገር<br>4. የለም	
L96	ስለኤች አይብ ዉጤቶን ለሌላ ሰዉ ነግረዉ ነበር?	1.አዎን 2. አይደለም 3. ፈቃደኛ አይደለሁም/መልስ የለም	2 ወደ 98
L97	ከነገረዎት ሰዉ ምን አይነት እርዳታ አግኝተዉ ነበር?	1.ምክር አገልግሎት 2.ገንዘብ 3.መረጃ<br>4.ሕክምና/መድኃኒት 5.ሌላ 6. ምንም	
L98	አሁን **<u>መጠይቁን ጨርሰናል</u>**።ምንአልባት ተጨማሪ አስተያየት ካሎት?<br>---		
L99	በጣም ከባድ ጥያቄዎችን ጠይቅያለሁ።ይህንን መነጋገራችን ምን እንዲሰማዎት አደረገ?<br>ምላሹን ይጻፉ?<br>1. ጥሩ/መልካም<br>2. መጥፎ/ጥሩ ያልሆነ<br>3. ተመሳሳይ/ ልዩነት የለም<br>**አመሰግናለሁ!**		
