# Supplementary material for: Intimate partner violence against women living with and without HIV, and the associated factors in Wolaita Zone, Southern Ethiopia: A comparative cross-sectional study
Source: PLoS One. 2019 Aug 23;14(8):e0220919. doi: 10.1371/journal.pone.0220919 (PMC6707594; doi:10.1371/journal.pone.0220919)
Supplement: S3 File — (PDF) [file pone.0220919.s003.pdf]

Oduwa Worakata, maayetuwanne pillgetta oyyishatta (Maacassa oyichiyo Oyyisha)

Oduwa Worakata, maayetuwanne pillgetta oyyishasearch

Gaalassa:--

Ayimala loo dë̈etti?, Tasuntayyi ------------------------Tanni mantta Koyira Maskale Mangistuyi kwuwaZulu- Natalle universitiyanni doctorettetta tamariyage kassi kasse Wolayita Soddo universitiyanni luxisiyaga ottiyo pilgettawu qofa shakanawu yaasi.. Ha manta gayyittanawu koyikko , entte demanawu sikiyya payidoyi (+251913177996 woyikko imellle: mengistu77@gmail.com). Entenna haa pillgetta yagnidoo gishawu bonchuwanni shobetidetta " Haa pilligettasika kusha qoffayi "**Nu Topianni Wolayita Zonniyan garsanni bari matta laggiyappe Echi Ayibiyara de"iyanne Echi Ayibiiyara bayinna macaa assa gakkiya shoochiya meeziyanne, aarra gaayitidabatubba** "

Ha pillgettawu huppe hallchooyi maccassa payattettanne ettu duussa meeziya tamarrannee qasika ettu heeran hannetiabba errannassa .

Nunni mullera 816 oyishsha Wolaitta Zonniyanni uddupuni payattetta naagiyo ketttatun de"iya maacassa oyichettes. Heegeka hagappe kaaliya marranni pollettessi. Nuni enttenna oyisha oyichanna,entte suntta xappokko . Entte ha pillgettan ishi gikko , haaga wursanayiyo 25-30 daqiqqa ekkes.

Haa pilgettassi haaggappe kaaliya kohoyi woyike injetenabbay de"annawu danddayees. Entte haa pilgettani gellidi, enttena gakkida qohuwa shakiyo wodde amarida injetenabbayi siyettanna dandaayees. Hegga gidikonne, darro macaassasi wozannaba laggiyara shakiyogge loo"o qaadda. Onni erri entte kaasse adhiida itta meeziya nuuyiyo odishshin yibbatisiyabbi dee"ikonne zooriyanni maadannasinne intte miiyiyanni eqanasi payattetta naagiya keetawu yeddana. Nunnika nubaggarra haa oyishsha oggi maakidi enttena zooranna. Ha pilgettaayika maadiyoge bari matta lagiyappe gakiya shochiya haanotasi nu heera hanotta demanasinne halcuwa poliyaa getusi qoppa imannasa.

Haa pilgettay UKZN assa boollani ossettiya pilgetta matta maabbarayi zaari xellidinne ottana maala maatta imidossona (Maayuwa payidoyi:---------------------------------).

Gelladanni mettoyi de"ikko woyikko oyishayi de"ikko haa pilgeetta ottiyagga maanta Meskele Mengistu silkiya payiduwani (+251913177996 Woyiko imelliya : mengistu77@gmail.com) UKZN assa bollani ottiyo pillgetta maabara demiyogee Haagappe garissara de"es.

Biomedical Research Ethics Administration

1. **Research Office, Westville Campus**
2. **Govan Mbeki Building**

Private Bag X 54001
Durban
4000

KwaZulu-Natal, South Africa

Tel: 27 31 2604769 - Fax: 27 31 2604609

Email: BREC@ukzn.ac.za

Haa pillgettanni entte gellanage entte shenne.Toppiyanni de"iya maacassawu entte meeze keehippe go"es. Haa pilgetta oyichishinni ayi woodiyanika agagana gikko dandayassa. Aggo gishawu neenni kasse go"ettiyobbatuppe attiyabbikka woyikko gaakiya qoheettikka baawa.

Haa pilligetta qooppa shaakko giishshawu aayibbinne emettiyabi baawa. Giddoshine daroo machasayi bantta ullubba baa lagiyaara hassayannawu lo"o qaada demessi.

Taani entteyo boonchuwanni ersiyoogge dee"ikko entte taasi oddobba ubba kossanni naaganna..Entte heerayinne entte suntayi haa duqittiyabatu giddo oyikkettiddi uttenaga pillgetta ottiya manttawayi erssesi. Nunni entta cengurssa duqannashinni ubbayika xuuranni oyiiqettesi. .Entte sunttayika ayii maziggabba bollanikka xafettenna. Ha entteppe nuni ekkiddi xaafiddo qooffa ubbayi xuurranni oyiqettiddi ossinne yootettena. Haa pillgettappe kiiyidi attamettiyabba entte suntta qoncissenna. Nunni enttenna ooyisha oyyichishin assi geelikko , harra cimiyobba haassayidi baallettanna.

Maayetuwa

Taanni (-----------------------------) Mantta Koyira Meskele Mengistu ottiyo "**Nu Topianni Wolayita Zonniyan garsanni bari matta laggiyappe ''Echi Ayibiyara de"iyanne Echi Ayibiiyara bayinna macaa assa gakkiya shoochiya meeziyanne, aarra gaayitidabatubba"** pillgettababba siyassi.

Ha piilgetta halchuwakka akeekkassi.

Tassi gellibeyinnabayi de"ikokka taanna koshshiyagga oyichadda erannawu kaddayi immettissi.Taanni qooncissiyogge , haa ooyishanni beetidogge mulerrakka ta sheeniyanna.

Haa pilgetta oyichishinni ayi woodiyanika agagana dandaayayissi. Aggo gishawu taanna kasse go"ettiyo xaaliya eehuwappe woyikko bonchuwappe attiya qohoyi baawa.

Haa pilgeettara gaayittidaganni taanna gaakiyaa qoohoyi de"ikko , hee baalassi kooshiyaa kaassa qaanxiyogga wooyikko xaaletiyogga do"iyabba siiyassi.

Geelladanni mettoyi de"ikko woyikko oyishayi de"ikko haa pilgeetta ottiyagga maanta Meskele Mengistu silkiya payiduwani (+251913177996 Woyiko imelliya : mengistu77@gmail.com)

Qaasikka taayiyo jujo ooyiishayi piilggiiya maanttawa boolli de"ikko haagaappe kaalliya xooqassa ooyichannagge qoonceettis.

Biomedical Research Ethics Administration

1. **Research Office, Westville Campus**
2. **Govan Mbeki Building**

Private Bag X 54001
Durban
4000

KwaZulu-Natal, South Africa

Tel: 27 31 2604769 - Fax: 27 31 2604609

Email: BREC@ukzn.ac.za

Oyishetida assatu paarama Date

---------------------------------- --

Markatu paramm

1. -- ---
2. --- --
3. Oyichiyaga suntan--------------------- ---

Oyisha IV: Quantitative Study

Kifiliiya 1. Essippettettanne yelletaba

Ma.P.	Oyiisha	Zaaruwa	Piiritte
	HIV pillgetta ayi malle?	1. ART talliya ekkiyaga 2. HIV bollanni bawa	
S1	Entte layitta appunne?	Kumeetta Laayittayi --------------------------	
S2	Entte deˈiyossayi awee?	1.kaattama 2. Gaatariya	
S3	Haaˈi enttee geeluwa ekuwabbay ayimale??	1. Ha''I azina gelassid/ Azinara deˈayissi →<br>2. Attumassara deˈayis gelabeyikke →<br>3. Haˈi ubbade tanara gayittiya attumayi desishini gelabeyikke →<br>4. Haˈi wodiyan gellabeyikke / attumassara de7ike.	S7<br>S7<br>S7
S4	Haggappe kasse gelada woyikko attumassara deˈada erayi?	1. Eee, gellassi<br>2. Eee, attumassara deˈasi , shin gellabeyikke<br>3. Kassi	
S5	Attumassara nessi wursettanni deˈiya gaytottettay birshettidetti, shaketidettiye , woyiko ne matta lagge hayiqide?	Birshettida..............................1<br>Shakettida/Meqisi .... 2 →<br>4. Azinayi Hayiqissi............3	S7
S6	Ha birshetta koyiro qattidayi nenne, ne kettawayi woyikko naˈayikka issippe mayettidetti?	Maccasiyo ....1<br>Keettawa ....2<br>Naˈayikka issippe ....3<br>Harrayi : ________________.....4	

S7	Ne deˮuwan azinna apputo gelladi/ woyiko attumassarra woqa deˮadi?	Gellidoga payidoyi/ Issippe de7idoge ------<br>Akkayi zaroyyi immikke---------------------8	
S8	Nennara benni entte gelletishin ne kettawassi harra maccassiya deˮayi?	Eee...1<br>Kashi..**2**<br>Hassayikke…………………………......3<br>Akkayi zaruwa immike 4	
S9	Nennara datti ayyo appun macassa deˮi?	Macca payidoyi ....<br>Akkayi/Zarroyi bawa 8	
S10	Nenni koyirro/naˮantto ………Macasse/ lagge?	Payiduwa/ daraja .... [ ][ ]<br>Zaruwa ixissi 8	
S11	Ha7i ne azinna nenni dooraddi, harra assayi neyo doridde, woyikko izawa doridde nenna?<br>Nenni dorennabba gidikko , haˮi nne kettawa onni nessi dooride?	Naˮayikka issippe dorrida...........**1**<br>Maccasiya dorasu..........................**2**<br>Maccase ketta assayi doorissi ....3<br>Kettawayi doorisi ....4<br>Azinna ketta assayi doorissi.................5<br>Wossiyo kettayi dorrisii........................6<br>Tanni erikke/ Akekike .. ……7	
S12	Haa kettawa gellanappe kasse /aˮi matta kettawa gellanawu nenni oyichadi?	Eee...1<br>Kashi...2<br>Akekkikke……………………………… .3<br>Zaruwa ixxisii……………. 4	
S13	Nee gellido ciiloshayi immettide?	Eee /cillosha ....1<br>Eee/ Maccasasi immiyo waga....................2<br>Akkayi ....**3**<br>Errike/Assayikke………………………… **4**	
S14	Maccassa ciiloshayi ubbayika qanxettide woyikko , sinttappe qanxettanawu deˮiyabbayi haˮikka deˮi?	1.Ubbayikka qanxettissi 2 .Kibbayi qanxettissi<br>3 . Ayibbikka qanxettibenna<br>4 .Tanni erikke /Skakikke	
S15	Neesoni deˮiyaa assa paayido wooqe?	-----------------------------------	

S16	Entte haayimanotte ayibe?	1.Ortodokisiya 4. katolikiya<br>2.Issillama 5. Woogaa amanuwa<br>3.Protestantiya 6.Haara	
S17	Entte sheeshshaa ayibbe?	.Wolaita 4.Gurage<br>2.Amaara 5.Oromoo<br>3. Dawuro 6. Haara	
S18	Entte Osoyi ayiibe?<br>(Maaccasasi)	1. So osuwa 2. Zal7ancha 3. tamariya<br>4. Kaawuwa/Kaawo gidenna 5. Qaane osancha<br>6. Haara-----------------------------	
S19	Nee keettawa osso ayibe?	1. Goshshancha 2. Kawo osancha 3. Zaldhancha<br>4. Qanne Osancha 5. Kaawo Osuwappe kaare<br>6. Haara	
S20	Kaalame timihirtiya tamaaradi?	0. --------------------Kashi<br>1.--------------------Eenno	Kaashi Giiko 111
S21	Entte wursido xooqa kiifille aapunne?	Wuursiddo kifiille.......................................	
S22	Nee keettawa kaalamiya timihitiya taamaride?	0. --------------------Kashi<br>1.--------------------Ee	If "no" GO to Q. 113
S23	Nee keetawa ogiido xooqa kiifille aappunne?	Wuursiddo xooqa kiifille----------------------	
S24	Entte ketta aginnanni demiyo mishayi/damoza woqqe ?	Toppiya birranni: ----------------------------------	

S25	Entte keettanni ………………: [Xaappa ‘1’ Liike gidikko & ‘0’ Kaashi Giddiko]<br><br>W1. Tellevission: |___| W2. Radoniya: |___|W3. Sattelayitte Diishiya: |___| W4. Mobilliya silkiya: |___| W5. Soo Silkiya: |___| W6. Xarapheza: |___| W7. Woonbara |___| W8. Piirashiya/puttuwa/shubuwa pirashiya osettida aliga: |__| <br>W9. Korrinttiya bashiya :|___| W10.Biskilittiya: |___| W11. Mottorira sakilliya/bajajiya W12. Pirijiya |___|W13. Electirikiya|___| W14.Tracteriyya/Makinna|___| W15. Gosha bitta/Kattama bitta:|___| W16.Budenna bashiya mittan kattiyaga: |___| W17. Eletrikiya midija

Kiifilliya II. Ne a7i matta laggiya/azinnabba qoppiyode , hagape garssada de7iyabbayi tumme?

Ma.P.	Oyiishaa	Eee	Kaashi	Errikke
D27	Nee laagiya woyikko daabuwa be‟enna maalla diigess?	1	2	8
D28	Entteso keettta assaara(yeelidageturra) gaayittenna maalla teqees?	1	2	8
D29	Neeni ubba woodde de‟iyosa eraanawu kooyesi?	1	2	8
D30	Neenna olligesi wooyikko dumma xeellessi?	1	2	8
D31	Haara attumassara hassayikko yiillottes?	1	2	8
D32	Amaannetaka giiddi ubbadde siiressi?	1	2	8
D33	Neehuppiyawu paayattetta demanayo baa sheeniya oyichaanaga kooyessi?	1	2	8

Kifiiliya III: Haagappe kaalidi de"iyage daaro maaccassa bollanni ha"i haziinnayi woyikko

Ma.P.	Oyiisha	A) Enno giddiko ''B'' kko bitte.Akkayi giddikko kaaliyagakko bitte		B) Haage adhdhida 12 aginnattu gaarssani haanide?(Enno giidiko"C" xalla oyichay. Akkayi giidiko"D" xalla oyicha)		C)) Haaga adhdhida 12 aginnattu gaarssani haanidogge issitto, amarida wode, woyikko daarotto? (''C'' zaaridi simiddi kaalliyagakko biitte)			D)12 aginaappe kaasse haagge haannidoyi issitoyi, amarida wode, Darro wode?		
		Ee	Akayi	Ee	Akayi	Issitto	Amaratto	Daarot to	Issitt o	Am ara tto	Daarot to
E34	Nenna caayidde woyikko neeyo ittaba siiyettanadann ottide?										
E35	Harra assa sinttani kawushide?										
E36	Erriyidi issibbaa neenna yaashisanawu otti(Lem.Waasidi woyiko yashisiyidi neenna xeelidde) ?										
E37	Nenna woyikko neenni dichiyoba yashiside?										

	Oyisha<br>**Nee keettawayi woyikko nee haarra lagge….**	A) Enno giddiko ‘’B’’ kko bitte.Akkayi giddikko kaaliyagakko bitte		B) Haage adhdhida 12 aginnattu gaarssani haanide?(Enno giidiko ‟C‟ xalla oyichay. Akkayi giidiko ‟D‟ xalla oyicha)		C)) Haaga adhdhida 12 aginnattu gaarssani haanidogge issitto, amarida wode, woyikko daarotto? (‘’C’’ zaaridi simiddi kaalliyagakko biitte)			D)12 aginaappe kaasse haagge haannidoyi issitoyi, amarida wode, Darro wode?		
		Eee	Akkayi	Eee	Akkayi	Issitto	Amaratto	Daarotto	Issitto	Amaratto	Daarotto
P38	Issibanni neena shociide woyikko issibayi qohannamalla caadidde?										
P39	Suugidde woyikko huuphiya neegga daapidde?										
P40	Bubuxide woyiko nenna qoohiya haarabanni shocidde?										
P41	Neenna kaakidde, goochide woyikko shodhide?										
P42	Yiiloyide woyikko eriiyide neenna xugiide ?										
P43	Zayiiyani yashiside woyikko heega go‟etidde, biilama woyikko harra maasariya neenna qoohannawu goo‟ettide?										

S.N.	Oyisha **Nee keettawa woyikko haara laage neenna …**	A) Enno giddiko ‘’B’’ kko bitte. Akkayi giddikko kaaliyagakko bitte		B) Haage adhdhida 12 aginnattu gaarssani haanide? (Enno giidiko"C" xalla oyichay. Akkayi giidiko"D" xalla oyicha)		C)) Haaga adhdhida 12 aginnattu gaarssani haanidogge issitto, amarida wode, woyikko daarotto? (‘’C’’ zaaridi simiddi kaalliyagakko biitte)			D)12 aginaappe kaasse haagge haannidoyi issitoyi, amarida wode, Darro wode?		
		Ee	Akayi	Ee	Akayi	Issitto	Amaratto	Daarotto	Issitto	Amaratto	Daarotto
S44	Ha"i nee keetawayi woyikko haara laagge baa wolkanni sugidi neenni koyenna asho gayittotettassi wa"iside?	1	2	1	2	1	2	3	1	2	3
S45	Neenna nee keettawa woyikko harra lage neenni koyennanni de"ishinne yellatishin asho gaakketta neenara polliede?	1	2	1	2	1	2	3	1	2	3
S46	Ne kaaha bashibaani nee keetawa woyikko haara laage issiba asho gakettaba ssi wolkanttide?	1	2	1	2	1	2	3	1	2	3

Ma.P.	Oyiisha	
P47	Bollanni gaakiya shochayi woyikko toshshee de ̎iyakko shaakka?<br>Osha payiduwa xeella 400-405.	1.Eee , bollanni gaakiya shochayi woyikko toshshee de ̎es<br>2.Akkayi, bollanni gaakiya shochayi woyikko toshshee baawa.
S48	Ashuwa gayittottetani yiya toshe de ̎ikko shaka?<br>Osha payiduwa xeella 500-502	1.Eee, Ashuwa gayittottetani yiya toshe de ̎es.<br>2. Akkayi, Ashuwa gayittottetani yiya toshe baawa.
I49	Abbe ixeetta giidaara errayi?	1.Eee<br>2.Akkayi If 2 Go to I52
I50	Entte abbe ixeetta gididi eriyoga odidetta. Abbe ixeetta giddirashi nee keetawa shocidi woyiko tochchidi erri?	1.Eee<br>2.Akayi<br>3.Errikke /Haasayikke If 2,3,4 Go to I53<br>4.Ixxissi/Zaaroyi baawa
I51	Haa ne keettawa tochetappe dendidaganni bosheshettara erayi?	1.Eee<br>2.Akkayi
I52	Neeni haa nee maata keetawappee woiyikko haara laagiiyappee bolla shoeetada, heegeka qanxeettada, xugeetada, meeqetta woyikko achaa me ̎ada haa nunni qomora haasayido gasuwappe denddidagan erayi?	| | Eee | Akay | |---|---|---| | 1. Qanxoyi,cadettoyi,satayi | 1 | 2 | | 2. Garettayi,uzuziyabbayi | 1 | 2 | | 3. Burqe, Madhiyappe shodetidabayi | | 2 | | 4. xugettayi | 1 | 2 | | 5. Gido gelida cachayi,suxiyabayi | 1 | 2 | | 6. Me7ida hayittayi, ayifiya qanxoyi | 1 | 2 | | 7. Meqoyi, meqetta meoyi | 1 | 2 | | 8. Acha meoyi | 1 | 2 | | 9. Harrabbayi | 1 | 2 |
I53	Nee de ̎uwanni , nee keetappe woyiikko haara laagiyaappe woyiisa mansunxaaddi?<br>Neenni heega issitto, naa ̎utto woyikko daaroto gaayi ?	1. Issito/Naa7utto<br>2. Daaroto(3-5) wode<br>3. Keehipe daroto (5 moolla)<br>4. Taa errikke / Haasayikke<br>5. Ixxisi / Zaaroyi baawa.
I54	Has this happened in the past 12 months?	1. Eee<br>2. Akkayi<br>3. Taa errikke / Haasayikke<br>4. Ixissi/ Zaaroyi baawa
I55	Hee neqohuwayo hakimya marpiya ekkadi?	1.Eee issitto issitto ekassi<br>2.Eee ubbattokka ekasi<br>3.Kashi ekabbeyike
I56	Hee quhuwasi assappe maduwa koyadda erayi?	1.Ee<br>2.Akayi

Maacassa bollanni gaakiya toshshiya

I57	Haa ne keettawayi nenna shociiyogaa oossi yottadi?	Onninne maddibbena.......................................1<br>Lagettusi..2<br>Yellidagetusi...3<br>Ishasi / Micheesi…... ..4<br>Awwa michesi/Awa ishassi ....5<br>Azinna keetta assassi…………. ....6<br>Natussi …...7<br>Shooruwassi...8<br>Pollisiyaasi..9<br>Hakimiyas/Payattetta nagiyagettusi.................10<br>Amanuwa kalletiyagetusi…............................ ..11<br>Zoriyagetusi…...12<br>Kawwo dirjittiya gidennagetussi/Maacassa mabarrassi…………………………………………………..13<br>Heera kalletiyagetusi .... 14<br>Higgiya mabbarawu ....15<br>Haaratusi(Qoncissa):____________________16
I58	Neenna maddida assi de'i?<br>Onne maddiddayi?<br>Ubba zaruwakka malattta: Onne Haarrayi madiddayi?	Onninne maddibbena.......................................1<br>Lagettusi..2<br>Yellidagetusi...3<br>Ishasi / Micheesi…... ..4<br>Awwa michesi/Awa ishassi ....5<br>Azinna keetta assassi…………. ....6<br>Natussi …...7<br>Shooruwassi...8<br>Pollisiyaasi..9<br>Hakimiyas/Payattetta nagiyagettusi.................10<br>Amanuwa kalletiyagetusi…............................ ..11<br>Zoriyagetusi…...12<br>Kawwo dirjittiya gidennagetussi/Maacassa mabarrassi………………………………………………….13<br>Heera kalletiyagetusi .... 14<br>Higgiya mabbarawu ....15<br>Haaratusi(Qoncissa):____________________16
W59	Neenni shoocettido wode neenna ashanau zaarada arra waretada woyikko nee huphiya ashioba ottaddi?<br>1.Eee<br>2.Akkayi<br>Enno giidikko: Apputo? Neeni heega issito woyikko naa'utto , daarotto	1. Issitto woyikko Naa'utto<br>2. Daarotto /Keehippe daarotto , /daaro woodiyanna<br>3. Taa errikee / Akeekike<br>4. Ixxissi / Zaaroyi baawa

	wooyikko daaro woode?	
W60	Neenni ne keetawa nenna bochena diishin aa ashuwa shochaddi, woyiko tochaddi?<br>1.Eee 2. Akkayi , Enno giidikko: Apputto? Neeni heega issito woyikko naaʽutto , daarotto wooyikko daaro woode?	1. Issitto woyikko Naaʽutto<br>2. Daarotto/Keehippe daarotto , /daaro woodiyanna<br>3. Taa errikee / Akeekike<br>4. Ixxissi / Zarro bawa

Maaccassa daabuwappe, haara etti eriyoo assappenne imaattappe gaakiya toshiya

MaP.	Oyiisha		Zarroyi immetidogaa kanchiya oyicha. Apputto heege annidde?		
			Issito Woyikko Naaʽutto	ımaaridda diyassa	Daarotto
L61	**15 layittappe siminni:** Ayyi assa giidikkoka (Haaʽi woyikko kaasse aziinnara deeʽiya maaccasaayi: Nee keetawappe haarayi / laagiyappe) nee boolla shochiciddi erri?<br>1.Eee<br>2. Akkayi, Eee giidikko : Onne heega nee bollani ottidde ?<br>Piilgitte :<br>Nee daboyishin?<br>Nee taamariyosan woyikko ottiyossan deʽiyabbi?<br>Laage woyikko shooroyi?<br>Immattayi woyikko haara assayi?	1.Onnikka baawa	1	2	3
		2. Awaa	1	2	3
		3. Keetaye aziina	1	2	3
		4.Keetanni deʽiya haara attumassa	1	2	3
		5. Keetanni deʽiya maaccassa	1	2	3
		6. Teacher	1	2	3
		7. Police/ soldier	1	2	3
		8. Keetan deʽiya attuma laage	1	2	3
		9. Soonni deʽiyaa maacca laggiyo	1	2	3
		10. Attuma Laagiya	1	2	3
		11. Immatta	1	2	3
		12. Osso ketta deʽiya haara assa	1	2	3
		13. Keesiya/Hayimanotiyaga	1	2	3
		14. Kaaletiyaga	1	2	3

Maaccassa daabuwappe, haara etti eriyoo assappenne imaattappe gaakiya toshiya

Ma.P.	Oyisha		Heegga ottiyowode laayitta neeyo wooqe?	Heega ottiya izzawa qaasi appunn laayitte?	Zarroyi immetidogaa kanchiya oyicha. Apputto heege annidde?		
					Issitto /Naa̎utto	Ammarida wodiyawu	Daaro woodiyawu
L62	**15 layitappe kaasee**, issi assi nenna haa keettanni asho gayittettotawu bochidakonne haassayayi,woyikko nenni kooyennanni de̎ishinni asho gayittetta ottanawu issibba ottide? Eee giddikko: Onne heegga ottidagge nee bollanni? Eee ottis gikko oyisha dooma: Taamare keettanni de̎iya asse? Laagge woyikko shooro? Haara asse haaga nee boollanni ottiday? Eee giddikko: Who did this to you?	1.Onnikka baawa			1	2	3
		2.waa			1	2	3
		3. Keetaye aziina			1	2	3
		4.Keetanni de̎iya haara attumassa			1	2	3
		5. Keetanni de̎iya maaccassa			1	2	3
		6. Teacher			1	2	3
		7. Police/ soldier			1	2	3
		8. Keetan de̎iya attuma laage			1	2	3
		9. Soonni de̎iyaa maacca laggiyo			1	2	3
		10. Attuma Laagiya			1	2	3
		11. Immatta			1	2	3
		12. Osso ketta de̎iya haara assa			1	2	3
		13. Keesiya/Hayimanotiyaga			1	2	3
		14. Kaaletiyaga			1	2	3

Ma.P.	**Oyiisha**	Zaaruwa	Xaalitte
	Neenii azzinaappe gaakiya tooshiyabba ayiippiyanni bee̎addi woyikko markatadda errayi?	1. Eee<br>2. Akkayi	

L64	Neenna nee bollaa bollanni woyikko ashuwa bollanni gaakiya shochayi gaakiyode?	1. Higawe giddida maaduwa kooyadassa<br>2. Hiigawee giidenna maaduwa kooyaddassa	
H65	Nee keettawayi mattoyiya ushsha uyii?	1. Ee<br>2. Akayi	
H66	Awude awude ushsha uyii? Ubbade, issitto issito, mullekka uyyenna?	1. Ubbade<br>2. Saminttan issitto woyikko nautto<br>3. Agginappe 1-3<br>4. Issito issitto<br>5. Tanni erikke<br>6. Ixxissi	

S.N	Oyisha	Response			Response
H68	Ne kettawa ushara gayittidaganni hagappe kalliya abba mettoyi gaakidde?<br>a) Mishsha mettoyi<br>b) Payattetta mettoyi<br>c) Keetta assara /laaggiyara oshayi<br>d) Maattayi de "iyogettura mettoyi (Uttelya gottatura/polissetura, h.h.m)<br>x) Haarata, qooncissa		Ee	Akkayi	
		A)Miisha metuwa	1	2	
		B) Payattetta metuwa	1	2	
		C) Keetta assara/lagiiyara osha	1	2	
		D) Maattayi de"iyogettura mettoyi (Uttelya gottatura/polissetura,	1	2	
		E) Harra	1	2	
L69	Ha "i nenna kasse oyidu samminttatu garssani uni"idabatta hassayana. Ha"i oyicha : Ne layittani ne shempuwa woranna gadda qooppada errayi ?	Eee.............1<br>Akkayi...............2<br>Akekkike ....3<br>Ixxisi/Zaaroyi bawa................4			
L70	Nee shipiyo woorannawu mallada errayi??	Eee..1<br>Akkayi ....2<br>Akkekike................................3<br>Ixxisi/Zaaroyi baawa................4			
H71	Ne matta wode keetawa/laggiya kondomiya go"eetossi gadda oyicha erayi?	Eee..1<br>Akayi..2<br>Akekikke...3			

		Ixxisi 4	
H72	Ne matta wode keetawa/laggiya kondomiya go"eettikke giddy ixxide?	Eee.. ...1<br>Akayi...2<br>Akekkike...3<br>Ixxisi...4	If 1 Go L74
H73	Kondomiya go"ettikke yagogga nessi ayibba oogiyanni yottide? Ubba zaaruwaka malatta	Go"ettikke yaggidi yottissi .... A<br>Waasissi/Yillotisi .... B<br>Shocannawu yashisisi .... C<br>Yedanawu/keetappe karre yedanawu gissi .... D<br>Tanna shocissi.............E<br>Kondomiyo xayisis /ekkisi ....F<br>Amannetakka yagidi motissi/Lo"o maccassa giidakka giisi......................... G<br>Ta bollanni micissi/sheneho giisi....... .. H<br>Hegge maadenna giisi ....I<br>Harrabba ________________________________ X	
L74	Tanni ha"i nenni kochoruwanni gayittiddo asho ngakettabba oyichanna. Koyiro neenni asho gayittottetta oottiyode nessi appunni layitte?	Kumetta layitta-----------------------------	
L75	Koyiro nenni asho gayitottetta ottiyode ayibbi nenna gaakide?<br>Ta maattana; Tanna amanttissi; Tanni cimettasi; Tannaa wolkanttisi; tanna dafarissi	Hege ta sheenne.........................1<br>Tanni amannettasi.......................2<br>Tanni cimettasi............................3<br>Tanyo wolqamisi..........................4<br>Tanni dafaretassi.........................5	
L76	Heegge onnara haanide?	Keetawa/lagiyara..........................1<br>Attuma siquwara...........................2<br>Astamariyara................................3<br>Awara/Taa keetta assara..............4<br>Tamare keettan heeraattumassara......5<br>Keetta assa laagiyara...................6<br>Taa dabuwaara............................7<br>Immattara/erenna assara...........8<br>Haarara....................................... 9	
L 77	Neenni arra asho gayitottetta ottiyode assi appunn layitte? Nenni ayo	Layitta [ ][ ]<br>Taappe daressi.............................1<br>Tagga malla..................................2<br>1-2 layitta tappe darressi...............3<br>3-5 layitta tappe daressi.................4<br>5-10 layitta taappe daress...............5<br>10 layitta taappe dareesi.................6<br>Zaaruwa ixxisi...................................9	
L78	Macca aassassi asho gaytottettasi de"iya atumassa qodayi issuwappe issuwanni dumattessi . Issi issi assassin issi laggeHaarassi qassi 2 woyikko arroyi, harrassi qassi daroyi, ubbaka 50 nne heegappe dariyaga. Nessi layittani qassi ayikkenna assa asho	Lagettu payidoyi.................... [ ][ ][ ]<br>Akekikke.......................................1<br>Ixxisi/ Zaaroyi bawaa 2	

	gayittetetta ottadi?<br>Koshiyabba gidiko pilga: Darooye gutte; Tanni like payiduwa koyikke.					
L79	Nee naʽattetta wode, ne ayiyya nne awanni shoʽettade?	Eee...1<br>Akkayi..2<br>Yellidagetti issippe deʽokonna......................3<br>Errikke………..4<br>Ixxissi…………………. 5				
L80	Naʽattettani , haa tochetta beʽaddi?	Eee...1<br>Akkayi..2<br>Tanni erikke…………………………………………..3<br>Ixxissi/Zaaroyi baawa…………………………..4				
H81	Nenni eriyode ne azinna ayiya ba keettawanni shoʽettade?	Eee...1<br>Akkayi ....2<br>Yellidagetti issippe deʽokonna………. .....3<br>Akekkikke…………………………………………..4<br>Ixxassi/Zaaroyi baawa……………………….5				
H82	Nee matta azinnayi/ lagge haa toshiya beʽidde?	Eee..1<br>Akkayi...2<br>Akkekkike...3<br>Zaaroyi bawa………………. 4				
H83	Nenni eriyode nne matta keettawa/lagge ubba wodde onnanninne ba keetta assappe shoettidi erri?	Eee..1<br>Akayi...2<br>Akkekike…………………………………….....3<br>Ixxisi/Zaaroyi bawa..............................4				
H84	Nenni eriwodiyappe dommidi ne keetaway harra assara warettidi eriyee?	Eee...............................1<br>Akayi ....2<br>Erikke/Akekike…………….3<br>Ixis/Zaaroyi baawa………. 4				
	Kiffiliya: **Barri kettayiyo shochanawu deʽiya**<br><br>Ha mabarran gidi harrani assassin bari so assassin dumma dumma qofayi deʽes. Qassika maccassawukka attumassau wogga gidiya eshayi deʽ3ss. He qooppa qashuwa taani nessi nababanna.Hegga qassi nenni maayettiyakonne maayettenako yootassa. Heegasika like woyikko like gidenna zaaroyi baawa.					
A85	Nee qoofanni , Keetawa barri maccassiyo shocannayiyo loʽoo gassoyi daannawu dandayi?		Yes	No	DK	
	aBarri keetawa ufayisiyattuwan soo ossuwa oottada wursana xaayiko?	A) Soo keetta	1	2	3	
	b) Barri keetawassi azazetanna xayiko	B) Azaazettennani xaayiko	1	2	3	
	c) Asho gayittetetawu ixissi giiko	C) Ashuwa gaayitottetta ixxikko	1	2	3	
	d) Assi gelaʽo laagge deʽiyakonne oyichiko?	D) Gelaʽo laagiya	1	2	3	
	e) Keetawa barikettayio amannetuku giidi siriko.	E) Siiriko	1	2	3	
	f) Keetayiya amannettenaga I demiyabba giidiko	F) Amannettenna	1	2	3	

A86	Nee qoofani, Geellida macassayi bari keetawassi asho gayitottetta ixxanawu dandayiyo ogge :<br>a) Maacassiya koyennani xaayikko<br>b) Izawi usha uyiyabba giidiko<br>c) Keetayiya uyiyabba giidiko<br>d) Keetawayi o toochiko<br>e) Keetawasi harra Haara corra maccssa lagge de7esi gada siiriko<br>f) Baari keetawassi basho gayittettappe oyikiya sakoyi/ HIV de"essi gaada siiriko	A) Koyennanni xaayiko<br>B) Usha uyiiko<br>C) Saaketiko<br>D) Toochiko<br>E) haara maccassa siriko<br>F) HIViya siiriko	1<br>1<br>1<br>1<br>1<br>1	2<br>2<br>2<br>2<br>2<br>2	3<br>3<br>3<br>3<br>3<br>3	
	Zariyagga nne I keetawaa					
H87	Nenninne ne keetawayi haagappe kaaliya huphe yohuwabba issippe haasayidetti:<br>a) Galla galla hannettidabatun<br>b) Galla ne bollanni hannidabatunni<br>c) Nenna mettidabatunni<br>d) A meettidabatunni	a) Kettawa gallassan<br>b) Nee gallassan<br>c) Ne mettuwanni<br>d) A mettuwanni	Yes<br><br>1<br>1<br>1<br>1	No<br><br>2<br>2<br>2<br>2	DN<br><br>3<br>3<br>3<br>3	
H88	Nenninne ne matta wode keetawayi haagappe asho gayittottetta wattidi ottannako, awude ottannako, apputte ottanako hassayidi eretti?	Ayininko……………………………………………………1<br>Issitto issitto……………………………………………2<br>Ubatto……………………. ……………………………3<br>Hassayidi erreko…………………………………………4<br>Ixxisi/Zaaroyi bawa……………………………………5				
H89	Ne matta wode azinnara de"iya gayitottetanni, arra wooqa wodiya warettidetti?<br>Hinno ayiniko ayiniko wode gayiye, issitto issitto woyike ubbade?	Ayininko……………………………………………………1<br>Issitto issitto……………………………………………2<br>Ubatto……………………. ……………………………3<br>Hassayidi erreko…………………………………………4<br>Ixxisi/Zaaroyi bawa……………………………………5<br>Warrettidi erokko--------------------------------------6				
L90	HIV maramarettadi?	Eee……………………………1<br>No………………………………2<br>Zaaruwa ixxissi………………….3				
	**HIV de"iyo assa kanchiya oyicha. Oyishsha L98 baa maccassayi HIVe bayinabba giidiko**					
H91	HIViya wuxettiya ne kettawassi /laagiyassi yootadi?	Eee………………………………………1<br>Akayi……………………………………2<br>Ixxissi/Zaroyi bawa…………………….3				If 1 Go L93
L92	Ne keetawayoo/laagiyayo nee HIVibiya wuuxettiya yottannawu halchayida de7ayi ?	YES……………………………………………………1<br>NO……………………………………………………2<br>REFUSED/NO ANSWER…………………………3				
L93	Ne keetawassi/laagiyassi nee HIv iya wuxettiya qoncissada yootannawu woqa wodiya ekidde?	Heezu gallassa gidoni…………………………………1<br>Issi samitta garsan……………………………………2<br>Issi agginna,,,,,,……………………………………………3<br>Heezu agginna ,,,,,, ……………………………………4				

		Ussupunni aginna..5<br>Ussupun agginappe bolla..................................6	
L94	HIV wuxettiya siyiddi makki simiddi nee keetawa /matta lagge besido eshayi ayi malle?	Tanna maddisi..1<br>Ta bollanni wassissi...2<br>Tanna zempississi...3<br>Tochisi/sho"isi..... ....4<br>Kawusha toshiya...5<br>Barri HIV tamarississi ....6<br>Ta asho gayitottetta tarikiya oyichisi...............7<br>Hakimiya/nursiya zoretta oyichisi....................8<br>Shocannawu yashisisi.....................................9<br>Ollidi aggiggannawu yashisissi.......................10<br>Tanna olliddi aggiyaagissi...11<br>Asho gayittettetta aggiyagissi.........................12<br>Harra maccassa ekkissi...................................13<br>Taani erike..14<br>I uppayittissi...15<br>Harrabba...16	
L95	Neeni qoopiyodde arranne nee keetawarra de"iya gaayittottetta kassegappe HIV wuxettiya yotta siminni lo"uwawu lamettideye ettawu lamettide?	Lo"uwawu..1<br>Iittawu...2<br>Ayibbawunne laamettibenna............................3	
L96	Nee HIV wuxettiya harra assawu yootadi?	Eee...1<br>Akkayi..2<br>Zaaroyi ixxissi...9	
L97	Ettappe aymalla maaduwa demaddi?	Zorriya...1<br>Miisha..2<br>Maraja..3<br>Xaaliya/kiininiya...4<br>Haarabba..5<br>Ayibbakka demabbeyikke..................................6	
	**Kiffiliya Uddufunna 9: Oyisha wursetta**		
L98	Ha"i nunni oyisha wurssida. Nenni immiyo qoopi haari de"i, gujannawu koyiyobbi haarabbi de"i?<br>--<br>--<br>--		
L99	Keehippe mettya oyishatta oyichassi. Ha oyishatunni neessi siyettidabbi ayibbe...?<br>Oyishettiya izawi imido zaaruwa mulliyakka xaappa?<br>____<br>**Wurssa**	Lo"o.................1<br>Iitta/lo"enna ....2<br>Issi malla/Dummabi bawa 3	

Ha oyiisha zaaranawu beettido giishawu keehippe Gaalatosi !
