# Supplementary material for: Intimate partner violence against women living with and without HIV, and the associated factors in Wolaita Zone, Southern Ethiopia: A comparative cross-sectional study
Source: PLoS One. 2019 Aug 23;14(8):e0220919. doi: 10.1371/journal.pone.0220919 (PMC6707594; doi:10.1371/journal.pone.0220919)
Supplement: S4 File — (PDF) [file pone.0220919.s004.pdf]

UNIVERSITY OF KWAZULU-NATAL™
INYUVESI YAKWAZULU-NATALI

28 September 2018

Mr MM Koyira (217982145)
School of Nursing and Public Health
College of Health Sciences
217082145@stu.ukzn.ac.za / meningstu77@gmail.com

**Protocol: Intimate partner violence against women living with and without HIV: Context and associated factors in Wolaita Zone, Ethopia**
**Degree: PhD** **BREC Ref No: BE387/18**

**EXPEDITED APPLICATION: APPROVAL LETTER**

A sub-committee of the Biomedical Research Ethics Committee has considered and noted your application received on 25 June 2018.

The study was provisionally approved pending appropriate responses to queries raised. Your response received on 12 September 2018 to BREC letter dated 27 July 2018 have been noted by a sub-committee of the Biomedical Research Ethics Committee. The conditions have now been met and the study is given **full ethics approval** and may begin as from 28 September 2018. Please ensure that site permissions are obtained and forwarded to BREC for approval before commencing research at a site.

This approval is valid for one year from **28 September 2018.** To ensure uninterrupted approval of this study beyond the approval expiry date, an application for recertification must be submitted to BREC on the appropriate BREC form 2-3 months before the expiry date.

Any amendments to this study, unless urgently required to ensure safety of participants, must be approved by BREC prior to implementation.

Your acceptance of this approval denotes your compliance with South African National Research Ethics Guidelines (2015), South African National Good Clinical Practice Guidelines (2006) (if applicable) and with UKZN BREC ethics requirements as contained in the UKZN BREC Terms of Reference and Standard Operating Procedures, all available at http://research.ukzn.ac.za/Research-Ethics/Biomedical-Research-Ethics.aspx.

BREC is registered with the South African National Health Research Ethics Council (REC-290408-009). BREC has US Office for Human Research Protections (OHRP) Federal-wide Assurance (FWA 678).

The sub-committee's decision will be noted by a full Committee at its next meeting taking place on **13 November 2018.**

We wish you well with this study. We would appreciate receiving copies of all publications arising out of this study.

Yours sincerely

Prof V Rambiritch
Chair: Biomedical Research Ethics Committee

cc postgraduate administrator: Ramlamm@ukzn.ac.za
Supervisor: khuzwayone@ukzn.ac.za
Co-supervisor: taylor@ukzn.ac.za
