# Supplementary material for: Intimate partner violence against women living with and without HIV, and the associated factors in Wolaita Zone, Southern Ethiopia: A comparative cross-sectional study
Source: PLoS One. 2019 Aug 23;14(8):e0220919. doi: 10.1371/journal.pone.0220919 (PMC6707594; doi:10.1371/journal.pone.0220919)
Supplement: S5 File — (PDF) [file pone.0220919.s005.pdf]

ወላይታ ሶዶ ዩኒቨርሲቲ
ወላይታ ሶዶ

Wolaita Sodo University
Wolaita Sodo

ም/ማ/አገ/ዘ/ም/ፕሬዚዳንት ጽ/ቤት
Vice President for Research and Community Services

ቁጥር/Ref.no--WSU 15/04/147

ቀን/ Date--12 Sep. 2018

To: Mr.Mengistu Meskele Koyira

Wolaita Sodo University

Subject: Ethical Clearance

Your research project proposal entitled"*Intimate Partner Violence against Women Living with and without HIV: Contexts and Associated Factors in Wolaita Zone, Ethiopia*" has been reviewed by the Institutional Review Board of Wolaita Sodo University for its ethical soundness, it is found to be ethically acceptable.

Thus, the Research and Community Service Vice President Office has awarded this ethical clearance for the stated study to be carried by Mr.Mengistu Meskele Koyira as of September 10, 2018.The investigators are expected to submit their research progress report to the Research and Community Service Vice President Office of the University.

Regards.

ዶ/ር ታከለ ታደሰ አዳፍሬ
Takele Tadesse Adafrie (PhD)
የምርምርና ማህበረሰብ አገልግሎት ም/ፕሬዚዳንት
Vice President for Research and Community Services

⊠138
Fax No 046-551 51 13
E-mil: wsuniv@ethionet.et
☎046-551 19 11
መልሱን ሲጽፉልን የእኛን ቁጥር ይጥቀ
In replying, please quote our ref. number.

Generated by CamScanner from intsig.com
